# Ancestral Origin and Dissemination Dynamics of Reemerging Toxigenic *Vibrio cholerae*, Haiti

**DOI:** 10.3201/eid2910.230554

**Published:** 2023-10

**Authors:** Carla N. Mavian, Massimiliano S. Tagliamonte, Meer T. Alam, S. Nazmus Sakib, Melanie N. Cash, Monika Moir, Juan Perez Jimenez, Alberto Riva, Eric J. Nelson, Emilie T. Cato, Jayakrishnan Ajayakumar, Rigan Louis, Andrew Curtis, V. Madsen Beau De Rochars, Vanessa Rouzier, Jean William Pape, Tulio de Oliveira, J. Glenn Morris, Marco Salemi, Afsar Ali

**Affiliations:** University of Florida, Gainesville, Florida, USA (C.N. Mavian, M.S. Tagliamonte, M.T. Alam, S.N. Sakib, M.N. Cash, J.P. Jimenez, A. Riva, E.J. Nelson, E.T. Cato, R. Louis, V.M. Beau De Rochars, J.G. Morris Jr., M. Salemi, A. Ali);; Stellenbosch University, Stellenbosch, South Africa (M. Moir, T. de Oliveira);; Case Western Reserve University School of Medicine, Cleveland, Ohio, USA (J. Ajayakumar, A. Curtis);; Les Centres GHESKIO, Port-au-Prince, Haiti (V. Rouzier, J.W. Pape);; Weill Cornell Medical College, New York, New York, USA (V. Rouzier, J.W. Pape);; University of KwaZulu-Natal, Durban, South Africa (T. de Oliveira);; Centre for the AIDS Programme of Research in South Africa (CAPRISA), Durban, South Africa (T. de Oliveira);; University of Washington, Seattle, Washington, USA (T. de Oliveira)

**Keywords:** Vibrio cholerae, cholera, bacteria, enteric infections, Haiti

## Abstract

The 2010 cholera epidemic in Haiti was thought to have ended in 2019, and the Prime Minister of Haiti declared the country cholera-free in February 2022. On September 25, 2022, cholera cases were again identified in Port-au-Prince. We compared genomic data from 42 clinical *Vibrio cholerae* strains from 2022 with data from 327 other strains from Haiti and 1,824 strains collected worldwide. The 2022 isolates were homogeneous and closely related to clinical and environmental strains circulating in Haiti during 2012–2019. Bayesian hypothesis testing indicated that the 2022 clinical isolates shared their most recent common ancestor with an environmental lineage circulating in Haiti in July 2018. Our findings strongly suggest that toxigenic *V. cholerae* O1 can persist for years in aquatic environmental reservoirs and ignite new outbreaks. These results highlight the urgent need for improved public health infrastructure and possible periodic vaccination campaigns to maintain population immunity against *V. cholerae*.

The ancient disease cholera remains a major public health threat in countries lacking safe drinking water, optimal sanitation, and preventive hygiene practices (*1*). In Haiti, which had not had a cholera outbreak in >100 years, toxigenic *Vibrio cholerae* O1 was detected in October 2010, after a major earthquake in January 2010 that destroyed much of the nation’s public health infrastructure. The first cholera epidemic wave in 2010 was likely caused by introduction of toxigenic *V. cholerae* O1 by peacekeeping troops from Nepal through contamination of Haiti’s Artibonite River by sewage outflows from the camp used by the peacekeeping contingent (*2*–*4*). Initial disease transmission was associated with exposure to water in the Artibonite River, then transmission throughout the country by human movement, tracking along major highways, and subsequent reentry of the microorganism into the aquatic environment (*5*–*8*). During October 2010–February 2019, more than 820,000 cases and nearly 10,000 cholera deaths were reported in Haiti (*9*).

Despite ongoing surveillance, no clinical cholera cases were reported in Haiti from February 2019 through early September 2022, leading to assumptions that cholera had been eradicated (*10*). However, on September 25, 2022, two *V. cholerae* infections were identified in the Port-Au-Prince metropolitan area (*11*), after which the outbreak rapidly spread across the country. By May 12, 2023, the epidemic had resulted in 41,944 suspected cases in all 10 departments of the country; 38,420 hospitalizations and 685 deaths were reported (*12*). Full genome cholera sequences from an isolate sampled on September 30, 2022 (*13*), and 16 additional isolates collected from Centre, Grand-Anse, and Ouest Departments during September 30–October 31, 2022 (*14*), showed that the 2022 strains were homogeneous and closely related to the clinical and environmental strains circulating in Haiti since 2010.

Our research group has been monitoring the cholera epidemic in Haiti since 2010 (*15*). Our work has highlighted the role of the aquatic environment in the initial 2010 epidemic and the ongoing evolution of *V. cholerae* strains collected as part of longitudinal collection of water samples from rivers and estuarine sites (*6*–*8*,*16*–*18*). Yet, the underlying driver of recurrent toxigenic *V. cholerae* O1 outbreaks in endemic settings remains highly debated. One scenario suggests that periodic introduction and transmission of new cholera strains within human populations is the major driver and that environmental aquatic reservoirs play little or no role, providing only a transient medium for the bacteria to pass from one host to the next (*19*,*20*). An alternative perspective argues that toxigenic *V. cholerae* O1, akin to other *Vibrio* spp., can persist in aquatic reservoirs with seasonal and occasional spillover into human populations and then exponentially spread from person to person (*21*,*22*). We used whole-genome sequencing and Bayesian phylogenetics and phylogeography to reconstruct the origin and dissemination dynamic of toxigenic *V. cholerae* reemergence during the 2022 epidemic in Haiti.

## Methods

For this study, we sequenced 42 clinical *V. cholerae* strains isolated during October–November 2022 and 48 previously unreported *V. cholerae* strains from clinical (n = 45) and environmental (n = 3) sources collected by our group during September 2017–June 2018, the last years of the previous epidemic wave. We used a Bayesian framework to construct phylogeny for those sequences, a large (n = 1,824) dataset of worldwide sequences, and publicly available sequences from strains isolated Haiti in 2022 (n = 17) and during 2010–2019 (n = 262, including 32 sequences from environmental isolates collected by our group) (*9*).

### Sample Collection and *V. cholerae* Isolation

To isolate toxigenic *V. cholerae* O1, stool samples were collected and immediately transported to the laboratory of the Groupe Haitien d’Étude du Sarcome de Kaposi et des infections Opportunistes (GHESKIO) or to the University of Florida (UF) laboratory in Haiti. The UF laboratory processed all environmental samples. Samples were enriched in alkaline peptone water or directly inoculated samples onto thiosulfate-citrate-bile-sucrose agar plates, or both, as described previously (*6*,*7*,*16*,*17*,*23*). We further characterized each isolate by serology and performed initial genetic characterization by using PCR techniques targeting toxigenic *V. cholerae* genes (*6*).

**Figure 1:**
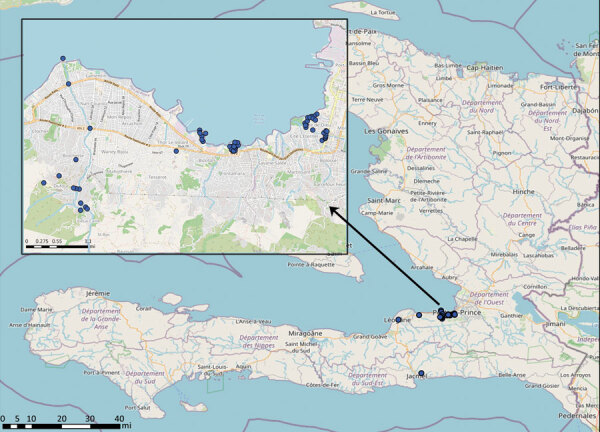
Selected sites of environmental sampling during used in a study of ancestral origin and dissemination dynamic of reemerging toxigenic *Vibrio cholerae*, Haiti. Blue dots indicate locations of environmental sampling sites for *V. cholerae* during 2012–2018. Inset shows detail of Port-au-Prince area sampling sites. Maps created by using OpenStreetMap (https://www.openstreetmap.org).

We initiated *V. cholerae* environmental studies in 2012, collecting water samples each month from a series of 17 fixed environmental sampling sites in rural river and estuarine areas in Gressier and Leogane (Figure 1). We previously reported isolation of *V. cholerae* from 10 (59%) of the 17 collection sites and 17 (8.6%) of 197 surface water samples in 2013–2014 (*6*,*7*). *V. cholerae* isolation was seasonal and associated with higher surface water temperatures and increased rainfall (*6*,*7*). We did not see a correlation between fecal coliform counts and *V. cholerae* isolation, suggesting that the environmental toxigenic *V. cholerae* O1 we isolated were autochthonous and not associated with fecal contamination at the collection site (*6*,*7*). We subsequently expanded site locations to include multiple sites in the Port-au-Prince region; sites in Jacmel, on the southern coastline of Haiti; and sites along the Artibonite River, where cholera was introduced in 2010 (Figure 1). We isolated a total of 32 toxigenic *V. cholerae* O1 bacteria from aquatic environmental sites during 2012–2018.

### Whole-Genome Mapping and SNP Calling

The UF Emerging Pathogens Institute (Gainesville, Florida, USA) performed high-quality full genome next-generation sequencing on 90 strains from 2017–2018 and 2022 by using previously described in-house protocols (*6*–*8*,*16*,*18*) (Appendix). We trimmed raw reads and genome assemblies from GenBank (https://www.ncbi.nlm.nih.gov/genbank) and the European Nucleotide Archive (ENA; https://www.ebi.ac.uk/ena) databases by using fastp version 0.22.0 (*24*). We analyzed reads by reference mapping to the 2010EL-1786 strain (GenBank accession nos. NC_016445.1 and NC_016446.1) as a reference for the Haiti dataset (*8*,*18*) or the N16961 strain (GenBank accession nos. NZ_CP028827.1 and NZ_CP028828.1) as reference for the global dataset (*19*,*20*). We used Snippy version 4.6.0 (https://github.com/tseemann/snippy) for mapping and variant calling and Gubbins version 3.2.1 (*25*) to scan consensus genome alignments for recombination. For the global dataset, we split the alignment into clusters we identified with fastBaps version 1.0.8 (*26*) before recombination analysis (Appendix).

### Phylogenetic Inference with Worldwide *V. cholerae* Dataset

We inferred a maximum-likelihood phylogenetic tree by using IQ-TREE (*27*) to compare *V. cholerae* O1 strains from Haiti, including isolates from the 2022 outbreak, with 1,824 worldwide cholera strains collected during 1957–2022. The global collection comprised strains from Europe (n = 22), the Americas (n = 593, excluding the Haiti strains), Asia (n = 465), Africa (n = 743), and Oceania (n = 1). Strains from the Americas included those from an outbreak in Argentina in the 1990s and an outbreak in Mexico during the 1990s–2013. Samples from Asia included strains from Bangladesh (1971–2011 and 2022), Nepal (1994, 2003, and 2010), and a wide range of strains from India collected during 1962–2017. The collection from Africa included strains from the 2015–2017 outbreak in the Democratic Republic of the Congo (*28*). Strains from the Middle East included strains from Yemen in 2017 (*29*). We determined the phylogenetic signal by using the likelihood mapping test in IQ-TREE (*27*). We used TreeTime (*30*) to obtain a maximum-likelihood tree scaled in time.

### Phylodynamic Inference and Phylogeography

We used a Bayesian framework to infer a posterior distribution of trees and estimate the time of the most common ancestor of the sampled sequences. We considered strict or uncorrelated relaxed molecular clock models and constant or Bayesian skyline plot demographic priors. We ran Markov chain Monte Carlo samplers for 500 million generations, sampling every 50,000 generations, which was sufficient to achieve proper mixing of the Markov chain, as evaluated by effective sampling size >200 for all parameter estimates under a given model. We used BEAST version 1.10.4 (*31*) to perform Bayesian calculations. We obtained a maximum clade credibility (MCC) tree from the posterior distribution of trees by using optimal burn-in with TreeAnnotator in BEAST. For publishing purposes, we visualized the MCC phylogeny in R (The R Foundation for Statistical Computing, https://www.r-project.org) by using the ggtree package (*32*).

We performed Bayesian phylogeographic analysis in BEAST version 1.10.4 (*33*) by using groups as a discrete trait, an asymmetric transition (migration) model, Bayesian skyline plot as demographic prior, and Bayesian stochastic search variable selection models. We considered rates yielding a Bayes factor (BF) >3 as well-supported diffusion rates (*34*) and BF >6 as decisive support (*35*), constituting the migration graph. We used DensiTree version 2 (R. Remco et al., unpub. data, https://doi.org/10.1101/012401) to graphically edit phylogenetic trees (Appendix).

## Results

### Epidemiology of 2022 Outbreak and Characterization of Toxigenic *V. cholerae* O1 Clinical Isolates

**Figure 2:**
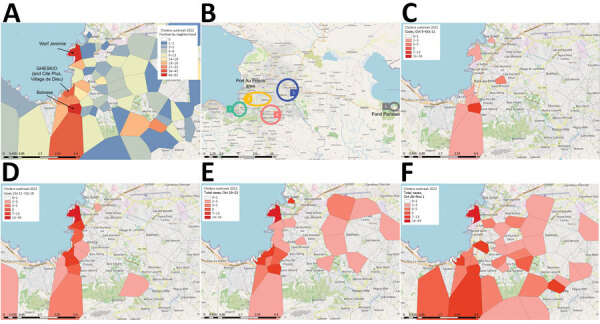
Temporal-spatial data of 2022 cholera cases in a study of ancestral origin and dissemination dynamic of reemerging toxigenic *Vibrio cholerae*, Haiti. Data were reported by GHESKIO CTC. A) Cumulative number of patients per Port-au-Prince neighborhood seen at the GHESKIO CTC during October–December 2022. B) Location of Fond Parisien site (no. 5 in gray circle) in relation to phylogeographic case groupings in Port-au-Prince neighborhoods: 1, GHESKIO area; 2, central eastern; 3, far eastern; 4, greater Pétion-Ville. C–F) Temporal and spatial distribution of the reported cholera cases by week: October 5–11 (C); October 12–18 (D); October 19–25 (E); October 26–November 1 (F). Maps created by using OpenStreetMap (https://www.openstreetmap.org). CTC, cholera treatment center; GHESKIO, Groupe Haitien d’Étude du Sarcome de Kaposi et des infections Opportunistes.

**Figure 3:**
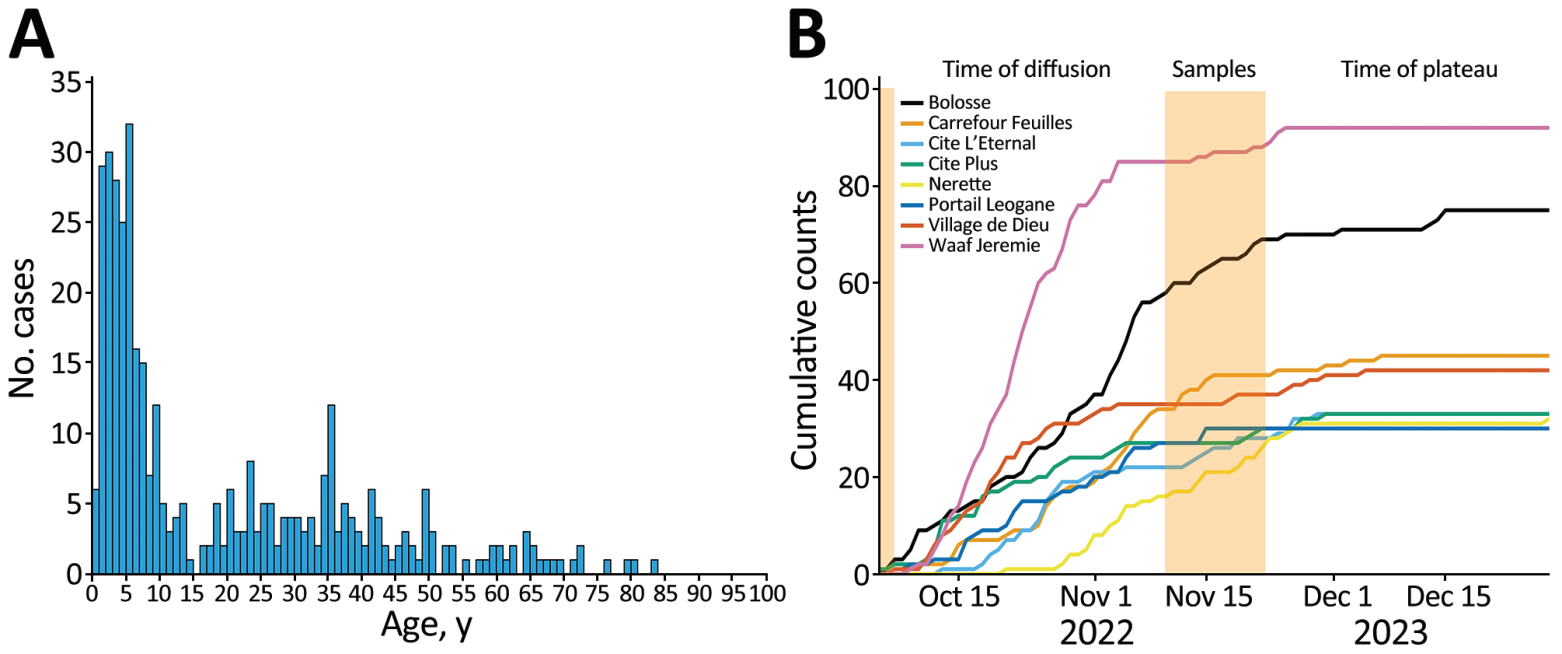
Characteristics of the 2022 cholera outbreak in Haiti based on reported positive cholera cases by GHESKIO. A) Age distribution of cases. B) Epidemiologic curves, by neighborhood, of cumulative cases over time from the GHESKIO cholera treatment center. Orange shading indicates sampling interval of this study. GHESKIO, Groupe Haitien d’Étude du Sarcome de Kaposi et des infections Opportunistes.

Our work focused on patients admitted to the GHESKIO cholera treatment center (CTC) in Port-au-Prince. GHESKIO CTC is near a portion of the city waterfront occupied by shantytowns (Figure 2, panel A), which were a key source of cases in the 2022 epidemic. Cases seen at GHESKIO CTC were concentrated among children (Figure 3, panel A), which aligned with national data reported by the Ministry of Public Health and Population in Haiti (*12*). Isolated *V. cholerae s*trains were susceptible to antimicrobial agents commonly used to treat cholera (*22*), including doxycycline, ciprofloxacin, and azithromycin (Appendix Table 1). However, ciprofloxacin susceptibility was borderline and molecular analysis showed a DNA gyrase mutation in *gyrA* (Ser83Ile) and *parC* (Ser85Leu) that was previously described in clinical isolates from Haiti (*36*,*37*).

Epidemiologic curves of cumulative cases from the GHESKIO CTC showed an exponential outbreak followed by a plateau phase at the beginning of November 2022 (Figure 3, panel B), as noted in national data (*12*). When we mapped the GHESKIO CTC case data to neighborhoods, the major initial hotspots of the epidemic were Bolosse, Village de Dieu, and Cite Plus, all of which are proximate to or southwest of GHESKIO, and Waaf Jeremy, north of GHESKIO (Figure 2, panel A). During October 2022, at the beginning of the epidemic, case foci clearly moved from an initial concentration along the coast to inland areas on a week-by-week basis (Figure 2, panels C–F).

We obtained sequence data for 42 toxigenic *V. cholerae* O1 strains collected during October 3–November 21, 2022: 40 from GHESKIO CTC and 2 from a clinic at Fond Parisien, which is in a rural area ≈30 miles east of Port-au-Prince and near the border with the Dominican Republic (Figure 2, panel B). All strains were serotype Ogawa and carried the genes for cholera toxin and other key genes associated with cholera pathogenicity and virulence (Appendix Table 2). All samples were collected between the end of the exponential phase and the beginning of the plateau phase of the epidemic (Figure 3, panel B). The UF institutional review board approved analysis and sequencing of the deidentified isolates.

We performed genomewide comparison of high-quality SNPs (SNPs) for those 42 sequences and 17 sequences from 2022 reported by others (*13*,*14*). Genomes were relatively homogenous and had a mean nucleotide distance in pair-wise comparisons of 1.26 high-quality SNPs, consistent with a single source introduction. However, 2022 cholera genomes from Haiti displayed 41–53 (median 24) high-quality SNP differences compared with the 2010EL-1786 reference strain. Compared with all previous strains from Haiti, a total of 5 mutations in coding segments of chromosome 1 were unique to the 2022 sequences, 1 synonymous and 4 nonsynonymous (Appendix Table 3). In addition, a 4-nt insertion caused a frameshift in the hypothetical gene HJ37_RS07360, resulting in a premature stop codon (Appendix Table 3).

As noted in prior publications (*13*,*14*), the maximum-likelihood phylogeny inferred from the genome-wide SNP alignment of 310 strains isolated in Haiti from 2010–2018, sequences from the 2022 outbreak (n = 59, including our 42 new sequences) and 1,824 worldwide reference sequences (Appendix Table 4) confirmed that the new cholera cases clustered within a well-supported (bootstrap >90%) monophyletic clade from Haiti (Appendix Figure 1). Those findings clearly demonstrate that the outbreak was caused by reignition of endemically circulating strains rather than outside introduction.

### Bayesian Phylogeography Dissemination during Early Outbreak Phases

**Figure 4:**
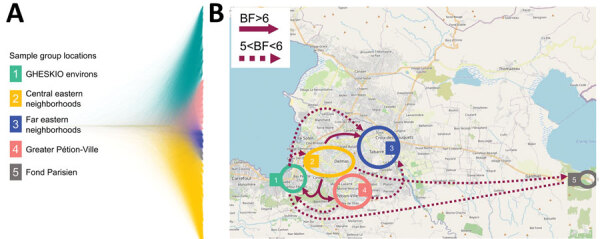
Bayesian phylogeography and dissemination patterns of reemerging toxigenic *Vibrio cholerae*, Haiti, during the 2022 outbreak. A) Bayesian DensiTree showing superimposed posterior distribution of trees inferred by the phylogeography analysis of *V. cholerae* full genomes from clinical cases sampled in Haiti during October 3–November 21. Colors indicate branches of samples grouped by location. B) Locations of case clusters delimited by colored circles. Solid- and broken-lined arrows indicate migration patterns among areas, as supported by Bayes factor and inferred from phylogeographic analysis by using a discrete trait asymmetric diffusion model. We considered rates yielding a BF >3 supported diffusion rates (*33*) and BF >6 decisive support (*34*), constituting the migration graph. Maps demonstrate major migrations of 5<BF<6 and BF >6. A list of all migrations supported by BF >3 are reported elsewhere (Appendix Table 5). BF, Bayes factor.

We reconstructed *V. cholerae* dispersal patterns for the 42 Haiti strains we sequenced and for which neighborhood of residence was known by using a Bayesian phylogeographic framework. Because of the short sampling time (October 3–November 21, 2022), we used a strict molecular clock and a fixed rate of 0.0179 SNP nucleotide substitutions per high-quality SNP site, which is similar to estimates obtained by previous studies (*8*,*16*,*18*), and the molecular clock analysis performed on our whole 2010–2022 Haiti dataset (Appendix). We used DensiTree to visualize the posterior distribution of trees obtained from phylogeography analysis to depict all probable migrations (Figure 4, panel A), from which we extracted migrations that were strongly supported by BFs of 5<BF<6 and BF>6 (Figure 4, panel B; Appendix Table 5). Cases included in the phylogeographic analysis tended to cluster in 4 general areas in Port-au-Prince (Figure 2, panel B; Figure 4, panel B). The snapshot of that phase of the outbreak provided by the phylogeography analysis is consistent with epidemiologic findings showing an epidemic hub within the Port-au-Prince administrative district and statistically significant (BF >6) migrations from group 2, corresponding to the central eastern neighborhoods of Port-au-Prince to other communes within the city and then to Fond Parisien (Figure 4, panel B). We also observed well-supported (5<BF<6) migrations within Port-au-Prince with origins in group 1, corresponding to the GHESKIO environs, and in group 4, corresponding to greater Pétion-Ville (Figure 4, panel B).

### Ancestral Origin of 2022 *V. cholerae* O1 Strains in Haiti

**Figure 5:**
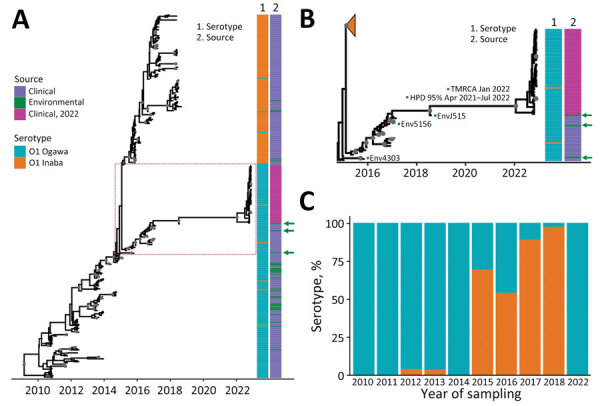
Inferred Bayesian phylogeny in a study of ancestral origin and dissemination dynamic of reemerging toxigenic *Vibrio cholerae*, Haiti. Phylogeny was inferred from 371 toxigenic *V. cholerae* O1 full genome clinical and environmental strains collected in Haiti during 2010–2022. A, B) Time-scaled phylogenies of *V. cholerae* serotypes inferred by enforcing a relaxed clock with Bayesian skyline demographic prior in BEAST version 1.10.4 (https://beast.community): A) Phylogeny of all isolates collected during 2010–2022. Dotted box denotes area detailed area shown in panel B. B) Detail of Ogawa clade from which the 2022 *V. cholerae* epidemic strains were derived. Gray circles indicate internal nodes supported by posterior probability >0.9. Branch lengths are scaled in time according to the x-axis. Time to MRCA of the 2022 Haiti isolates is shown at the node. Heatmaps denote clinical or environmental source and O1 serotype Ogawa or Inaba of the strains. Green arrows indicate the position of environmental strains basal to major clades. The collapsed orange clade refers to the monophyletic Inaba clade. Numbered green dots represent environmental *V. cholerae* O1 Ogawa isolates collected in Haiti; 2 were isolated from Jacmel Estuary, EnvJ515 in 2018 and Env4303 in 2015; Env5156 was isolated from a river in Leogane in 2016. C) Percentage of Ogawa and Inaba serotype isolates from samples collected in Haiti per year. HPD, high posterior density; MRCA, most recent common ancestor.

Toxigenic *V. cholerae* O1 strains sampled in Haiti during 2010–2014 were all Ogawa serotype, except for occasional sporadic Inaba strains. However, beginning in 2015, Inaba became the dominant clinical serotype, and by 2018 virtually all sampled clinical strains were serotype Inaba (*8*,*17*,*18*). During that time, toxigenic Ogawa strains were still detected from environmental sources (Figure 5; Appendix Figure 2, panels A, B). In contrast, all 2022 clinical strains were serotype Ogawa (Appendix Figure 2, panel C); thus, we sought to test the hypothesis that the new outbreak was linked to Ogawa strains persisting in the environment. Strong phylogenetic and temporal signals were detected in the whole dataset from Haiti, including clinical and environmental cholera samples collected during 2010–2022 (Appendix Figure 3). We inferred Bayesian MCC trees according to different molecular clock models and demographic priors (Appendix). To select the best fitting model, we compared path sampling and stepping-stone marginal likelihood estimates for each model pair by BF (Appendix Table 6). The MCC tree showed high support (posterior probability >0.9) for the 2022 strains sharing a most recent common ancestor (MRCA) with EnvJ515 environmental Ogawa strain (Figure 5, panels A, B), which was sampled in 2018 at a site on the Jacmel Estuary on Haiti’s southeastern coast. Path sampling and stepping-stone model testing also showed that although the phylogeny model enforcing monophyly with environmental strain EnvJ515 had a lower posterior probability than the unconstrained tree, the BF between the 2 models was not statistically significant (Appendix Table 6). Enforcing monophyly of the 2022 clade with other Ogawa or Inaba strains sampled in 2018 always resulted in a BF decisively supporting the null hypothesis of common ancestry between the 2022 strains and EnvJ515 (Table).

**Table:** Bayesian hypothesis testing of monophyly used in a study of ancestral origin and dissemination dynamic of reemerging toxigenic *Vibrio cholerae*, Haiti*

Monophyly testing†	lml(ML)_SS_‡	Ln(BF)_SS_§	lml(ML)_PS_‡	Ln(BF)_PS_§
Monophyly enforced with Ogawa 2014–2018 clade	–1,985.9	95.3	–1,988.4	94.5

MCC tree 2022 Haiti clade monophyletic with EnvJ515	–1,890.6		–1,893.9	
Monophyly enforced with Inaba 2018 clade	–1,987.9	97.3	–1,990.0	96.1
MCC tree 2022 Haiti clade monophyletic with EnvJ515	–1,890.6		–1,893.9	

*BF, Bayes factor; lml, log marginal likelihood; Ln, log; MCC, maximum clade credibility; ML, maximum-likelihood; SS, stepping-stone; PS, path sampling. †The test compares the MCC tree topology in Figure 5, panel B (H_0_), between 2022 environmental strains and 2018 environmental strain ENV515. Alternative topologies were obtained by enforcing monophyly with other strains (H_A_). ‡log of marginal likelihood estimates, lml(MLE), obtained by SS or PS by using a strict molecular clock and a Bayesian skyline demographic prior, with or without enforcing monophyly between the 2022 Haiti strains and different clinical strains. §log of BF comparing the null (H_0_) and alternative (H_A_) hypotheses.

The time to MRCA of 2022 outbreak strains was January 2022 (95% high posterior density interval of April 2021–July 2022), sharing a common ancestor with EnvJ515 in July 2018 (95% high posterior density interval May–July 2018) (Figure 5, panel B). Five high-quality SNPs in chromosome I and 1 in chromosome II differentiate the 2022 outbreak strains from EnvJ515; all are nonsynonymous mutations, and 2 affect hypothetical proteins (Appendix Table 7). We mapped an additional SNP difference in an intergenic region of chromosome II (Appendix Table 7). Although our focus was on EnvJ515, 2 other environmental Ogawa strains appear at the base of both the Ogawa and Inaba clades in the MCC tree: Env5156, which was isolated in 2016 from Leogane, on the north coast of the southern peninsula; and Env4303, which was isolated in 2015 from the Jacmel estuary where EnvJ515 was isolated (Figure 5, panel B). The 2022 outbreak strains and EnvJ515, Env5156, and Env4303 shared 24 high-quality SNPs, among which 16 are in coding segments, 9 cause a nonsynonymous change, and 2 cause a frameshift (Appendix Table 8). Those high-quality SNPs are a subset of a total of 41 that EnvJ515, Env5156, and Env4303 have in common, highlighting the link between the 2022 outbreak strains and the Ogawa strains persisting in the aquatic environment since 2015 (Appendix Table 9). During 2015–2018, a total of 4 Inaba strains were isolated from environmental sites in Gressier and Carrefour (Figure 1). However, phylogenetic analysis showed those strains were intermixed with concurrently isolated Inaba clinical strains and were not closely related to the 2022 Ogawa clinical strains or 2015–2018 Ogawa environmental isolates (Figure 5, panel A).

## Discussion

Our epidemiologic observations and phylogeographic analysis focused on cholera patients admitted to the GHESKIO CTC. Patient access to the GHESKIO CTC might have been influenced by transportation issues and local disruptions created by gang warfare in the city. Nonetheless, our overall observations support the hypothesis that the 2022 cholera outbreak in Haiti radiated from a single hub in Port-au-Prince, then spread exponentially, and was not caused by introduction of multiple *V. cholerae* strains. Of note, Bayesian hypothesis testing showed that the 2022 cholera strains shared a MRCA with an environmental lineage circulating in 2018. The location at the base of both Inaba and Ogawa clades of 2 other environmental Ogawa strains isolated in 2015 and 2016 is consistent with an established persistent environmental foci of this toxigenic *V. cholerae* O1 subclade (Figure 5, panels A, B). Of particular note, we isolated Ogawa strain Env4303 from the Jacmel estuary sampling site in 2015 and subsequently isolated EnvJ515 from the same site 3 years later, when virtually all clinical toxigenic *V. cholerae* O1 strains were serotype Inaba from a clearly distinct subclade. Our prior in vitro studies have demonstrated that toxigenic *V. cholerae* strains can survive in nutrient-poor aquatic environments for >700 days (*38*). Although the ecologic or strain factors driving persistence of *V. cholerae* strains within environmental reservoirs remain to be fully elucidated, persistence and subsequent spillover of strains from environmental foci into human populations in Haiti is supported not only by this study but also by prior phylodynamic studies from our group (*8*).

Jacmel, where EnvJ515 was isolated, is a popular local beach resort with easy access to Port-au-Prince and numerous restaurants, bars, and hotels lining the waterfront. Ten days before the first cholera case was reported September 25, 2022, Haiti had catastrophic flooding in the aftermath of hurricane Fiona (*39*), providing an ideal setting for environmental spillover of *V. cholerae* into water and food systems. Spread of the epidemic strain likely was further advanced by an abrupt interruption of the water supply by the national water company related to gang warfare and political unrest (*40*,*41*). That interruption resulted in an inability to provide potable water to shantytown areas of Port-au-Prince, a key area where the epidemic was identified. The shantytown areas are characterized by high-density, informal buildings and heavily polluted, open-air drainage channels coming from the city, which does not have a formal sewerage system or sewage treatment facilities. Those channels also drain water from the surrounding mountains and flow through the shantytown community into the harbor. Two major drainage channels pass through the areas near GHESKIO and Waaf Jeremy, areas that had particularly high case counts in the early weeks of the epidemic (Figure 2, panels C–F). Considering the local challenges with water and sanitation, introduction of *V. cholerae* into those areas, whether via the drainage channels or through human movement, almost certainly led to the emergence of initial cholera spatial hotspots and subsequent epidemic disease.

During the 2022 epidemic, infection rates appeared to be substantially higher among children 0–9 years of age, which is consistent with a lack of immunity to *V. cholerae* among this age group because they were not exposed to clinical cases in the preceding 3–4 years. Of note, clinical cases in the 2015–2019 outbreak almost exclusively were caused by *V. cholerae* Inaba serotype, and field-based studies suggest that initial Inaba infections protect against subsequent Ogawa infections (*42*). Thus, issues with cross-protection between the 2 serotypes and waning cholera immunity in the general population might have led to increased susceptibility to infection.

From a prevention standpoint, mathematical models we previously developed indicated that cholera eradication in Haiti will be difficult without substantive improvements to drinking water and sanitation infrastructure and that a clear potential for recurrence of epidemic disease from environmental reservoirs exists (*8*,*43*). Our previous modeling also underscored the potentially critical role that mass cholera vaccination can play in controlling epidemics (*44*). Although oral killed cholera vaccine has been used successfully in targeted campaigns in Haiti (*45*,*46*), efforts have not been made to immunize the entire country or to develop a long-term vaccination strategy. A major focus of prevention efforts in Haiti has been implementation of rapid response teams that go to homes of cholera patients and use sanitation and chlorination of household water to try to minimize transmission within households (*47*). Those efforts clearly are needed, but the ongoing risk for recurrent outbreaks from environmental reservoirs urges more action.

In summary, our data support the concept that a previously circulating Ogawa lineage served as the ancestor of *V. cholerae* strains that reemerged during the 2022 cholera outbreak in Haiti, suggesting a crucial link to the aquatic ecosystem. Links to environmental reservoirs documented in this study highlight the urgent need for overall improvements in public health infrastructure and water sanitation in Haiti and potential need for periodic mass vaccination campaigns to maintain protective levels of population immunity.

Appendix Additional information on ancestral origin and dissemination dynamic of reemerging toxigenic *Vibrio cholerae*, Haiti.
