# Supplementary material for: Ancestral Origin and Dissemination Dynamics of Reemerging Toxigenic Vibrio cholerae, Haiti
Source: Emerg Infect Dis. 2023 Oct;29(10):2073–82. doi: 10.3201/eid2910.230554 (PMC10521621; doi:10.3201/eid2910.230554)
Supplement: Appendix — Additional information on ancestral origin and dissemination dynamic of reemerging toxigenic Vibrio cholerae, Haiti. [file 23-0554-Techapp-s1.pdf]

Article DOI: https://doi.org/10.3201/eid2910.230554

EID cannot ensure accessibility for supplementary materials supplied by authors. Readers who have difficulty accessing supplementary content should contact the authors for assistance.

Ancestral Origin and Dissemination Dynamic of Reemerging Toxigenic *Vibrio cholerae*, Haiti

Appendix

Additional Methods

Antibiogram Assay

The susceptibilities of two early isolates (strains VCN3833 and VCN3834) from adult patients seen at the GHESKIO Cholera Treatment Center in Port-au-Prince on October 3rd and 4th 2022, were tested against antimicrobial agents using a Kirby-Bauer Disk Diffusion method following Clinical and Laboratory Standards Institute (CLSI) guidelines, as described previously (*1*) (Appendix Table 1).

Sample Collection, Sources, and Serotypes of *Vibrio cholerae* Strains from Haiti

A GIS database was constructed to map the dissemination of *Vibrio cholerae* cases in Haiti for three periods of time (2010–2016, 2017–2018, and 2022) (Appendix Figure 3). The data were obtained from the Pan American Health Organization (PAHO; https://ais.paho.org/phip/viz/ed_colera_casesamericas.asp). We collected in an Excel spreadsheet the coordinates of *V. cholerae* cases that contained additional information such as sampling location, date, source, and serotype, which was saved as a point shapefile using QGIS 3.28.3. The number of case "points" were analyzed for each department and pie charts created to display the serotype percentages. A population density layer (30 seconds arc ≈1 km of resolution) was applied in the background to visualize the spatial demographic data (people per square kilometer) in Haiti in 2022 from the Worldpop database (https://www.worldpop.org).

Whole-Genome Mapping and High-Quality SNP Calling

In this study, we analyzed a total of 371 *V. cholerae* O1 strains from Haiti collected from cholera patients and aquatic environmental reservoirs: 262 were from our previous BioProject (no. PRJNA510624) (*2*,*3*); 48 new strains were from 2018 (45 clinical and 3 environmental strains); and 59 new isolates were from patients in the 2022 outbreak, 44 new samples obtained in this study, and 17 publicly available sequences (*4*,*5*) (Appendix Table 4). All new strains (n = 90) from 2018 and 2022 were subjected for high-quality full genome next-generation sequencing (NGS) using previously described in-house protocols (*1*–*3*,*6*,*7*). Bacterial genomic DNA extraction was performed as previously described (*2*). Sample library construction was performed using the Nextera XT DNA Library Preparation Kit (Illumina) according to the manufacturer's instructions. Whole-genome sequencing on all isolates was performed by using the MiSeq Reagent Kit V3 for 600 cycles on the Illumina MiSeq System (Illumina). Raw reads and genome assemblies were downloaded from the NCBI and ENA databases; read quality assessment and trimming was done with fastp v.0.22.0 (*8*); data were analyzed by reference mapping, using the 2010EL-1786 strain (accession nos. NC_016445.1 and NC_016446.1) as a reference, as previously described (*2*,*3*). The Snippy v 4.6.0 pipeline (https://github.com/tseemann/snippy) was used to generate synthetic reads from genome assemblies, mapping to the reference genome, and call variants. Variant calling thresholds FreeBayes (E. Garrison et al., unpub. data, https://arxiv.org/abs/1207.3907) were set as minimum 10× site coverage, minimum mapping quality 60, and minimum 90% base concordance. Individual vcf files were merged using bcftools v.1.15 (*9*,*10*). Consensus genome alignments were scanned for recombination with Gubbins v.3.2.1 (*11*). For the global dataset, the alignment was first split into clusters identified with fastBaps v.1.0.8 (*12*) before recombination analysis. Fasta files manipulation was performed with seqkit v.2.0.0 (*13*) and Biostrings R package v.2.58 (2020); parsimony informative sites for phylogenetic analyses were extracted from consensus genome alignments using MegaX v.10.0.3 (*14*). Intra- and in-between group nucleotide distance was calculated using MegaX v.10.0.3 (*14*).

Phylogenetic Inference with Cholera Worldwide Dataset

We inferred a maximum likelihood (ML) phylogenetic tree using IQ-TREE (*15*) to compare *V. cholerae* O1 strains from Haiti, including the isolates from the 2022 outbreak, with worldwide cholera strains collected during 1957–2022 (n = 1,824), including strains from Europe

(n = 22), the Americas (n = 593, excluding the Haiti strains), Asia (n = 465), Africa (n = 743), and Oceania (n = 1). Samples from the Americas included strains from the outbreak in Argentina in the 1990s and in Mexico from the 1990s up to 2013. Samples from Asia include strains from Bangladesh (1971–2011 and 2022), Nepal (1994, 2003, and 2010), as well as a wide range of strains from India (1962–2017). The collection of African and Middle Eastern strains included samples from the recent outbreak in the Democratic Republic of Congo (M.T. Alam et al., unpub. data, https://doi.org/10.1101/2021.07.30.21261389) and Yemen in 2017 (*16*). Phylogenetic signal was determined using likelihood mapping test in IQ-TREE (*15*). A maximum-likelihood tree scaled in time was obtained with TreeTime (*17*).

Bayesian Framework for Phylodynamic Inference and Hypothesis Testing

We investigated the phylogenetic relationships of our 42 new clinical Haitian strains sampled during October 3–November 21, 2022 to: 1) 17 other new strains from Haiti collected in 2022 (*4*,*5*); 2) 48 new toxigenic *V. cholerae* O1 strains isolated in Haiti between September 2017 and June 2018 also sequenced in this study; and 3) a total of 262 toxigenic *V. cholerae* O1 strains previously isolated from clinical and environmental samples in Haiti (*2*,*3*) (Appendix Table 4). Phylogenetic signal was determined using likelihood mapping test in IQ-TREE (*15*) and temporal signal was estimated by plotting the root-to-tip divergence using TempEst (*18*). The Bayesian framework was used to infer a posterior distribution of trees and estimate the time of the most common ancestor (TMRCA) of the sampled sequences. Different molecular clock models (strict or uncorrelated relaxed molecular clock) and demographic priors (constant or Bayesian Skyline Plot) were considered. We also compared different tree topologies where monophyly was enforced between the 2022 Haiti monophyletic clade and specific environmental strains sampled in 2018, to test the hypotheses of common ancestry. Markov chain Monte Carlo (MCMC) samplers were run for 500 million generations, sampling every 50,000 generations, which was sufficient to achieve proper mixing of the Markov chain, as evaluated by effective sampling size (ESS) >200 for all parameter estimates under a given model. Hypothesis testing for best molecular clock, demographic model, and monophyly was performed by obtaining marginal likelihood estimates (MLEs) via path sampling (PS) and stepping-stone (SS) methods for each model to be compared, followed by the calculation of the Bayes Factor (BF), i.e., the ratio of the of the null (H_0) and the alternative hypothesis (H_A) MLEs (*19*–*22*), where lnBF<0 indicates support for H_0; lnBF<2, difference barely worth mentioning; 2<lnBF<6, strong support

for H_A, and *ln*BF>6, decisive support for H_A (*23*). The best model was strict clock with Bayesian skyline demographic prior (Appendix Table 6). Bayesian calculations were carried out with BEAST v1.10.4 software package (*20*). The maximum clade credibility (MCC) tree was obtained from the posterior distribution of trees using optimal burn-in with TreeAnnotator in the BEAST package. The MCC phylogeny was manipulated in R using the ggtree package (*24*) for publishing purposes.

Bayesian Phylogeography

Of the strains for which we had detailed sampling location information (Figure 1, panel B), nine strains were from nearby neighborhoods to the southwest of GHESKIO (group 1), 16 from neighborhoods to the east of GHESKIO (group 2), three from neighborhoods around Pétion-Ville (group 3), nine from Tabarre/Croix Des Bouquets (group 4), and two from Fond Parisien commune in the Ouest department near the border with the Dominical Republic (group 5). Strains from Rubin et al. (*4*) and Walters et al. (*5*) could not be incorporated in this analysis since they did not have the precise location made publicly available. Bayesian phylogeographic analysis was performed with BEAST v1.10.4 (*25*) using the groups as a discrete trait, asymmetric transition (migration) model, Bayesian skyline plot as demographic prior, and Bayesian stochastic search variable selection (BSSVS) models. Given the short sampling time interval of the Haitian sequences, we enforced a strict molecular clock with a fixed rate of 0.0179 SNP nucleotide substitution/hqSNP site, similar to previous estimates (*7*), and based on the molecular clock analysis of the whole (2010 −2022) Haitian dataset. We considered rates yielding a BF >3 as well supported diffusion rates (*26*), and BF>6 decisive support (*23*) constituting the migration graph. We showed diffusion rates with BF>5 in the figure and report al values in Appendix Table 5. Trees were edited graphically in DensiTree v2 (R. Remco et al., unpub. data, https://doi.org/10.1101/012401), available from https://www.cs.auckland.ac.nz/~remco/DensiTree. Xml files are available upon request.

References

1. Alam MT, Weppelmann TA, Longini I, De Rochars VM, Morris JG Jr, Ali A. Increased isolation frequency of toxigenic *Vibrio cholerae* O1 from environmental monitoring sites in Haiti. PLoS One. 2015;10:e0124098. PubMed https://doi.org/10.1371/journal.pone.0124098

2. Mavian C, Paisie TK, Alam MT, Browne C, Beau De Rochars VM, Nembrini S, et al. Toxigenic *Vibrio cholerae* evolution and establishment of reservoirs in aquatic ecosystems. Proc Natl Acad Sci U S A. 2020;117:7897–904. PubMed https://doi.org/10.1073/pnas.1918763117

3. Paisie TK, Cash MN, Tagliamonte MS, Ali A, Morris JG Jr, Salemi M, et al. Molecular basis of the toxigenic *Vibrio cholerae* O1 serotype switch from Ogawa to Inaba in Haiti. Microbiol Spectr. 2023;11:e0362422. PubMed https://doi.org/10.1128/spectrum.03624-22

4. Rubin DHF, Zingl FG, Leitner DR, Ternier R, Compere V, Marseille S, et al. Reemergence of Cholera in Haiti. N Engl J Med. 2022;387:2387–9. PubMed https://doi.org/10.1056/NEJMc2213908

5. Walters C, Chen J, Stroika S, Katz LS, Turnsek M, Compère V, et al. Genome sequences from a reemergence of *Vibrio cholerae* in Haiti, 2022 reveal relatedness to previously circulating strains. J Clin Microbiol. 2023;61:e0014223. PubMed https://doi.org/10.1128/jcm.00142-23

6. Alam MT, Weppelmann TA, Weber CD, Johnson JA, Rashid MH, Birch CS, et al. Monitoring water sources for environmental reservoirs of toxigenic *Vibrio cholerae* O1, Haiti. Emerg Infect Dis. 2014;20:356–63. PubMed https://doi.org/10.3201/eid2003.131293

7. Azarian T, Ali A, Johnson JA, Mohr D, Prosperi M, Veras NM, et al. Phylodynamic analysis of clinical and environmental *Vibrio cholerae* isolates from Haiti reveals diversification driven by positive selection. MBio. 2014;5:e01824–14. PubMed https://doi.org/10.1128/mBio.01824-14

8. Chen S, Zhou Y, Chen Y, Gu J. fastp: an ultra-fast all-in-one FASTQ preprocessor. Bioinformatics. 2018;34:i884–90. PubMed https://doi.org/10.1093/bioinformatics/bty560

9. Li H. A statistical framework for SNP calling, mutation discovery, association mapping and population genetical parameter estimation from sequencing data. Bioinformatics. 2011;27:2987–93. PubMed https://doi.org/10.1093/bioinformatics/btr509

10. Danecek P, Bonfield J, Liddle J, Marshall J, Ohan V, Pollard M, et al. Twelve years of SAMtools and BCFtools. Gigascience. 2021;10:giab008.

11. Croucher NJ, Page AJ, Connor TR, Delaney AJ, Keane JA, Bentley SD, et al. Rapid phylogenetic analysis of large samples of recombinant bacterial whole genome sequences using Gubbins. Nucleic Acids Res. 2015;43:e15. PubMed https://doi.org/10.1093/nar/gku1196

12. Tonkin-Hill G, Lees JA, Bentley SD, Frost SDW, Corander J. Fast hierarchical Bayesian analysis of population structure. Nucleic Acids Res. 2019;47:5539–49. PubMed https://doi.org/10.1093/nar/gkz361

13. Shen W, Le S, Li Y, Hu F. SeqKit: a cross-platform and ultrafast toolkit for FASTA/Q file manipulation. PLoS One. 2016;11:e0163962. PubMed https://doi.org/10.1371/journal.pone.0163962

14. Kumar S, Stecher G, Li M, Knyaz C, Tamura K. MEGA X: molecular evolutionary genetics analysis across computing platforms. Mol Biol Evol. 2018;35:1547–9. PubMed https://doi.org/10.1093/molbev/msy096

15. Nguyen LT, Schmidt HA, von Haeseler A, Minh BQ. IQ-TREE: a fast and effective stochastic algorithm for estimating maximum-likelihood phylogenies. Mol Biol Evol. 2015;32:268–74. PubMed https://doi.org/10.1093/molbev/msu300

16. Weill F-X, Domman D, Njamkepo E, Almesbahi AA, Naji M, Nasher SS, et al. Genomic insights into the 2016-2017 cholera epidemic in Yemen. Nature. 2019;565:230–3. PubMed https://doi.org/10.1038/s41586-018-0818-3

17. Sagulenko P, Puller V, Neher RA. TreeTime: Maximum-likelihood phylodynamic analysis. Virus Evol. 2018;4:vex042. PubMed https://doi.org/10.1093/ve/vex042

18. Rambaut A, Lam TT, Max Carvalho L, Pybus OG. Exploring the temporal structure of heterochronous sequences using TempEst (formerly Path-O-Gen). Virus Evol. 2016;2:vew007. PubMed https://doi.org/10.1093/ve/vew007

19. Baele G, Lemey P, Bedford T, Rambaut A, Suchard MA, Alekseyenko AV. Improving the accuracy of demographic and molecular clock model comparison while accommodating phylogenetic uncertainty. Mol Biol Evol. 2012;29:2157–67. PubMed https://doi.org/10.1093/molbev/mss084

20. Drummond AJ, Rambaut A. BEAST: Bayesian evolutionary analysis by sampling trees. BMC Evol Biol. 2007;7:214. PubMed https://doi.org/10.1186/1471-2148-7-214

21. Dittrich D, Leenders RTAJ, Mulder J. Network autocorrelation modeling: a Bayes factor approach for testing (multiple) precise and interval hypotheses. Sociol Methods Res. 2019;48:642–76. https://doi.org/10.1177/0049124117729712

22. Rouder JN, Speckman PL, Sun D, Morey RD, Iverson G. Bayesian *t* tests for accepting and rejecting the null hypothesis. Psychon Bull Rev. 2009;16:225–37. PubMed https://doi.org/10.3758/PBR.16.2.225

23. Xie W, Lewis PO, Fan Y, Kuo L, Chen MH. Improving marginal likelihood estimation for Bayesian phylogenetic model selection. Syst Biol. 2011;60:150–60. PubMed https://doi.org/10.1093/sysbio/syq085

24. Yu GC, Smith DK, Zhu HC, Guan Y, Lam TTY. GGTREE: an R package for visualization and annotation of phylogenetic trees with their covariates and other associated data. Methods Ecol Evol. 2017;8:28–36. https://doi.org/10.1111/2041-210X.12628

25. Drummond AJ, Suchard MA, Xie D, Rambaut A. Bayesian phylogenetics with BEAUti and the BEAST 1.7. Mol Biol Evol. 2012;29:1969–73. PubMed https://doi.org/10.1093/molbev/mss075

26. Lemey P, Rambaut A, Drummond AJ, Suchard MA. Bayesian phylogeography finds its roots. PLOS Comput Biol. 2009;5:e1000520. PubMed https://doi.org/10.1371/journal.pcbi.1000520

Appendix Table 1. Antimicrobial susceptibility test performed on *Vibrio cholerae* strains isolated from Haiti, 2022*

		Zone of inhibition per strain, mm		
Antimicrobial agent, µg	Abbreviation	VCN3833	VCN3834	Interpretation
Cephalothin, 30	KF	18	20	S
Ceftriaxone, 30	CRO	25	25	S
Cloramphenicol, 30	C	15	15	I
Cefixime, 5	CFM	20	21	S
Erythromycin, 15	E	16	15	I
Amikacin, 30	AK	15	16	I
Cefotaxime, 30	CTX	26	27	S
Ciprofloxacin, 5†	CIP	22	21	S
Azithromycin, 15	AZM	18	19	S
Streptomycin, 10	STR	7	7	R
Ceftazidime, 30	CAZ	23	23	S
Sulfisoxazole, 1,000	G	7	7	R
Sulphonamide, 1,000	SUL	7	7	R
Doxycycline, 30	DO	20	20	S
Ampicillin, 10	AMP	11	12	R
Sulphamethoxazole-trimethoprim, 25	SXT	7	7	R
Nalidixic acid, 30	NA	7	7	R
Tetracycline, 30	TE	22	22	S
Gentamycin, 120	CN	23	21	S

*Susceptibility testing performed by using standard disc diffusion assay. I, Intermediate; R, Resistant; S, Susceptible.
†Ciprofloxacin, a commonly used antimicrobial agen in Haiti exhibited a borderline sensitivity against *V. cholerae* strains (VCN3833 and VCN3834) with zone of inhibition (in diameter [mm]) found 22 and 21, respectively compared to standard upper threshold of the antibiotic (≥21 mm)

Appendix Table 2. Major pathogenicity islands and critical genes found in 2022 Ogawa *Vibrio cholerae* O1 strains, the 2018 Ogawa *V. cholerae* O1 strain (EnvJ515) isolated from the aquatic environment in Jacmel, Haiti, and the 2011EL-1786 reference strain

Pathogenicity island or gene	Description	Chromosome	Nucleotide bp, start–end
tcpA	Toxin coregulated pilin	I	367950–368624
ctxB	Cholera enterotoxin subunit B	I	1041238–1041612
wbeT	RfbT related protein	I	2687324–2688226
ideA	ICE encoded DNase	I	152890–153573
gyrA	DNA gyrase A subunit	I	807201–809885
parC	Topoisomerase subunit A	I	2073463–2075748
VPI-1	Vibrio pathogenicity Island	I	350786–391625
VPI-2	Vibrio pathogenicity Island	I	1367093–1424290
VSP-1	Vibrio pathogenicity Island	I	2600611–2614636
VSP-2	Vibrio pathogenicity Island	I	2947311–2961303

Appendix Table 3. Mutations unique to the 2022 *Vibrio cholerae* clinical isolates compared with all previous strains isolated in Haiti*

Gene or locus tag	Chr	Genome position	Mutation, nt	Mutation, aa	Protein ID, function
moaA	1	571,070	C to A	Thr310Asn	Molybdenum cofactor biosynthesis protein A (hypoxic growth)
fadJ	1	594,746	C to T	Ala517Val	Long chain fatty acid metabolism
mfd	1	1,503,970	T to C	Val709Ala	SOS response protein in response to sub-inhibitory level of antibiotic concentration
HJ37_RS07360	1	1,658,252	C to CGGCG	Ala74fs	
rpsG	1	2,803,192	A to G	Glu63Glu	30 S ribosomal protein S7

*aa, amino acid; Chr, chromosome; fs, frameshift; ID, identification; nt, nucleotide.

Appendix Table 4. Metadata and accession numbers of toxigenic *Vibrio cholerae* O1 strains used in a study ancestral origin and dissemination dynamic of reemerging toxigenic *V. cholerae*, Haiti*

Name	Serogroup	Source	Date decimals	Date uncertainty	SRA/ENA accession no.
Referent	O1 Ogawa	Clinical	2010.772603	0	NC_016445, NC_016446
AA-142	O1 Ogawa	Clinical	2010.854795	0	JSDO00000000
AA-143	O1 Ogawa	Clinical	2010.854795	0	JSCC00000000
AA-144	O1 Ogawa	Clinical	2010.854795	0	JSSV00000000
AA-145	O1 Ogawa	Clinical	2010.854795	0	JSSW00000000
AA-146	O1 Ogawa	Clinical	2010.854795	0	JSSX00000000
AA-147	O1 Ogawa	Clinical	2010.854795	0	JSSY00000000
AA-148	O1 Ogawa	Clinical	2010.854795	0	JSSZ00000000
AA-150	O1 Ogawa	Clinical	2010.854795	0	JSTA00000000
AA-151	O1 Ogawa	Clinical	2010.854795	0	JSTB00000000
AC5418–5	O1 Inaba	Environ	2018.191781	0	SAMN33739740 biosample accession no.
env-131	O1 Ogawa	Environ	2012.472678	0	JSTC01000061.1
env-326	O1 Ogawa	Environ	2012.653005	0	JSTG00000000
Env4926	O1 Ogawa	Environ	2016.65847	0	SRR23863573
Env5156	O1 Ogawa	Environ	2016.743169	0	SRR23863572
Env6956	O1 Inaba	Environ	2018.153425	0	SRR23863571
env-90	O1 Ogawa	Environ	2012.393443	0	JSTI00000000
env-94	O1 Ogawa	Environ	2012.393443	0	JSTK00000000
EnvJ515	O1 Ogawa	Environ	2018.556164	0	SRR23863570
GC-4740	O1 Ogawa	Clinical	2022.854795	0	SAMN33964162
GC-4741	O1 Ogawa	Clinical	2022.857534	0	SAMN33964163
GC-4744	O1 Ogawa	Clinical	2022.857534	0	SAMN33964164
GC-4746	O1 Ogawa	Clinical	2022.857534	0	SAMN33964165
GC-4751	O1 Ogawa	Clinical	2022.857534	0	SAMN33964166
GC-4755	O1 Ogawa	Clinical	2022.857534	0	SAMN33964167
GC-4786	O1 Ogawa	Clinical	2022.857534	0	SAMN33964168
GC-4789	O1 Ogawa	Clinical	2022.857534	0	SAMN33964169
GC-4791	O1 Ogawa	Clinical	2022.857534	0	SAMN33964170
GC-4792	O1 Ogawa	Clinical	2022.857534	0	SAMN33964171
GC-4823	O1 Ogawa	Clinical	2022.871233	0	SAMN33964172
GC-4824	O1 Ogawa	Clinical	2022.871233	0	SAMN33964173
GC-4828	O1 Ogawa	Clinical	2022.871233	0	SAMN33964174
GC-4832	O1 Ogawa	Clinical	2022.871233	0	SAMN33964175
GC-4834	O1 Ogawa	Clinical	2022.871233	0	SAMN33964176
GC-4835	O1 Ogawa	Clinical	2022.871233	0	SAMN33964177
GC-4837	O1 Ogawa	Clinical	2022.871233	0	SAMN33964178
GC-4838	O1 Ogawa	Clinical	2022.871233	0	SAMN33964179
GC-4868	O1 Ogawa	Clinical	2022.871233	0	SAMN33964180
GC-4872	O1 Ogawa	Clinical	2022.871233	0	SAMN33964181
GC-4900	O1 Ogawa	Clinical	2022.873973	0	SAMN33964182
GC-4902	O1 Ogawa	Clinical	2022.873973	0	SAMN33964183
GC-4903	O1 Ogawa	Clinical	2022.873973	0	SAMN33964184
GC-4905	O1 Ogawa	Clinical	2022.873973	0	SAMN33964185
GC-4907	O1 Ogawa	Clinical	2022.873973	0	SAMN33964186
GC-4908	O1 Ogawa	Clinical	2022.873973	0	SAMN33964187
GC-4941	O1 Ogawa	Clinical	2022.873973	0	SAMN33964188
GC-4943	O1 Ogawa	Clinical	2022.873973	0	SAMN33964189
GC-4944	O1 Ogawa	Clinical	2022.873973	0	SAMN33964190
GC-4978	O1 Ogawa	Clinical	2022.887671	0	SAMN33964191
GC-4979	O1 Ogawa	Clinical	2022.887671	0	SAMN33964192
GC-4980	O1 Ogawa	Clinical	2022.887671	0	SAMN33964193
GC-4981	O1 Ogawa	Clinical	2022.887671	0	SAMN33964194

Name	Serogroup	Source	Date decimals	Date uncertainty	SRA/ENA accession no.
GC-4985	O1 Ogawa	Clinical	2022.887671	0	SAMN33964195
GC-4994	O1 Ogawa	Clinical	2022.887671	0	SAMN33964196
GC-4996	O1 Ogawa	Clinical	2022.887671	0	SAMN33964197
GC-4999	O1 Ogawa	Clinical	2022.887671	0	SAMN33964198
GC-5000	O1 Ogawa	Clinical	2022.887671	0	SAMN33964199
H22_SRR22351617	O1 Ogawa	Clinical	2022.745205	0	SRR22351617
HC-07	O1 Ogawa	Clinical	2012.303279	0	JSTL00000000
HC-08	O1 Ogawa	Clinical	2012.303279	0	JSTM00000000
HC-10	O1 Ogawa	Clinical	2012.303279	0	JSTN00000000
HC-11	O1 Ogawa	Clinical	2012.382514	0	JSTO00000000
HC-12	O1 Ogawa	Clinical	2012.382514	0	JSTP00000000
HC-15	O1 Ogawa	Clinical	2012.382514	0	JSTQ00000000
HC-16	O1 Ogawa	Clinical	2012.382514	0	JSTR00000000
HC-17	O1 Ogawa	Clinical	2012.382514	0	JSTS00000000
HC-18	O1 Ogawa	Clinical	2012.382514	0	JSTT00000000
HC-19	O1 Ogawa	Clinical	2012.387978	0	JSTX00000000
HC-21	O1 Ogawa	Clinical	2012.382514	0	JSTU00000000
HC-22	O1 Ogawa	Clinical	2012.382514	0	JSTV00000000
HC-24	O1 Ogawa	Clinical	2012.382514	0	JSTW00000000
HC-31	O1 Ogawa	Clinical	2012.467213	0	JSTZ00000000
HC-32	O1 Ogawa	Clinical	2012.467213	0	JSUA00000000
HC-33	O1 Ogawa	Clinical	2012.467213	0	JSUB00000000
HC-34	O1 Ogawa	Clinical	2012.467213	0	JSUC00000000
HC-35	O1 Inaba	Clinical	2012.467213	0	JSUD00000000
LC-2746	O1 Ogawa	Clinical	2022.873973	0	SAMN33964160
LC-2747	O1 Ogawa	Clinical	2022.876712	0	SAMN33964161
SRR770779	O1 Ogawa	Clinical	2010.8	0	SRR770779
SRR771214	O1 Ogawa	Clinical	2010.8	0	SRR771214
SRR771222	O1 Ogawa	Clinical	2010.8	0	SRR771222
SRR771360	O1 Ogawa	Clinical	2010.79726	0	SRR771360
SRR771582	O1 Ogawa	Clinical	2010.8	0	SRR771582
SRR771645	O1 Ogawa	Clinical	2010.80274	0	SRR771645
SRR772254	O1 Ogawa	Clinical	2010.80274	0	SRR772254
SRR772256	O1 Ogawa	Clinical	2010.854795	0	SRR772256
SRR772892	O1 Ogawa	Clinical	2011.115068	0	SRR772892
SRR772893	O1 Ogawa	Clinical	2011.427397	0	SRR772893
SRR773027	O1 Ogawa	Clinical	2011.435616	0	SRR773027
SRR773028	O1 Ogawa	Clinical	2011.668493	0	SRR773028
SRR773104	O1 Ogawa	Clinical	2011.668493	0	SRR773104
SRR773107	O1 Ogawa	Clinical	2011.668493	0	SRR773107
SRR773175	O1 Ogawa	Clinical	2011.717808	0	SRR773175
SRR773179	O1 Ogawa	Clinical	2011.69589	0	SRR773179
SRR773315	O1 Ogawa	Clinical	2011.775342	0	SRR773315
SRR773317	O1 Ogawa	Clinical	2011.775342	0	SRR773317
SRR773321	O1 Ogawa	Clinical	2011.778082	0	SRR773321
SRR773389	O1 Ogawa	Clinical	2011.778082	0	SRR773389
SRR773393	O1 Ogawa	Clinical	2011.778082	0	SRR773393
SRR773397	O1 Ogawa	Clinical	2012.196721	0	SRR773397
SRR773656	O1 Ogawa	Clinical	2010.79726	0	SRR773656
SRR773657	O1 Ogawa	Clinical	2010.838356	0	SRR773657
SRR773658	O1 Ogawa	Clinical	2010.838356	0	SRR773658
SRR773660	O1 Ogawa	Clinical	2010.90411	0	SRR773660
SRR8364252	O1 Ogawa	Environ	2013.484932	0	SRR8364252
SRR8364253	O1 Ogawa	Environ	2013.484932	0	SRR8364253
SRR8364254	O1 Ogawa	Environ	2013.731507	0	SRR8364254
SRR8364255	O1 Ogawa	Environ	2014.413699	0	SRR8364255
SRR8364256	O1 Ogawa	Environ	2014.328767	0	SRR8364256
SRR8364257	O1 Ogawa	Environ	2013.89863	0	SRR8364257
SRR8364258	O1 Ogawa	Environ	2013.484932	0	SRR8364258
SRR8364259	O1 Inaba	Environ	2015.909589	0	SRR8364259
SRR8364260	O1 Inaba	Environ	2015.909589	0	SRR8364260
SRR8364261	O1 Ogawa	Environ	2015.70137	0	SRR8364261
SRR8364262	O1 Ogawa	Clinical	2013.654795	0	SRR8364262
SRR8364263	O1 Ogawa	Clinical	2013.657534	0	SRR8364263
SRR8364264	O1 Ogawa	Clinical	2013.690411	0	SRR8364264
SRR8364265	O1 Ogawa	Clinical	2013.649315	0	SRR8364265
SRR8364266	O1 Ogawa	Clinical	2013.690411	0	SRR8364266
SRR8364267	O1 Ogawa	Clinical	2013.706849	0	SRR8364267
SRR8364268	O1 Ogawa	Clinical	2013.709589	0	SRR8364268

Name	Serogroup	Source	Date decimals	Date uncertainty	SRA/ENA accession no.
SRR8364269	O1 Ogawa	Clinical	2013.665753	0	SRR8364269
SRR8364270	O1 Ogawa	Clinical	2013.665753	0	SRR8364270
SRR8364271	O1 Ogawa	Clinical	2013.715068	0	SRR8364271
SRR8364272	O1 Inaba	Clinical	2016.628415	0	SRR8364272
SRR8364273	O1 Ogawa	Clinical	2016.622951	0	SRR8364273
SRR8364274	O1 Ogawa	Clinical	2016.887978	0	SRR8364274
SRR8364275	O1 Inaba	Clinical	2016.92623	0	SRR8364275
SRR8364276	O1 Inaba	Clinical	2016.724044	0	SRR8364276
SRR8364277	O1 Ogawa	Clinical	2016.704918	0	SRR8364277
SRR8364278	O1 Inaba	Clinical	2016.680328	0	SRR8364278
SRR8364279	O1 Ogawa	Clinical	2016.642077	0	SRR8364279
SRR8364280	O1 Ogawa	Clinical	2016.822404	0	SRR8364280
SRR8364281	O1 Ogawa	Clinical	2013.484932	0	SRR8364281
SRR8364282	O1 Inaba	Clinical	2016.795082	0	SRR8364282
SRR8364283	O1 Inaba	Clinical	2016.814208	0	SRR8364283
SRR8364284	O1 Inaba	Clinical	2016.855191	0	SRR8364284
SRR8364285	O1 Inaba	Clinical	2016.887978	0	SRR8364285
SRR8364286	O1 Inaba	Clinical	2016.830601	0	SRR8364286
SRR8364287	O1 Ogawa	Clinical	2016.852459	0	SRR8364287
SRR8364288	O1 Ogawa	Environ	2013.726027	0	SRR8364288
SRR8364289	O1 Ogawa	Environ	2013.649315	0	SRR8364289
SRR8364290	O1 Inaba	Clinical	2017.156164	0	SRR8364290
SRR8364291	O1 Inaba	Clinical	2017.147945	0	SRR8364291
SRR8364292	O1 Inaba	Clinical	2017.147945	0	SRR8364292
SRR8364293	O1 Inaba	Clinical	2017.139726	0	SRR8364293
SRR8364294	O1 Ogawa	Environ	2013.726027	0	SRR8364294
SRR8364295	O1 Ogawa	Environ	2013.726027	0	SRR8364295
SRR8364296	O1 Inaba	Clinical	2017.394521	0	SRR8364296
SRR8364297	O1 Inaba	Clinical	2017.405479	0	SRR8364297
SRR8364298	O1 Ogawa	Environ	2013.821918	0	SRR8364298
SRR8364299	O1 Ogawa	Environ	2013.821918	0	SRR8364299
SRR8364300	O1 Inaba	Clinical	2017.378082	0	SRR8364300
SRR8364301	O1 Inaba	Clinical	2017.380822	0	SRR8364301
SRR8364302	O1 Inaba	Clinical	2017.389041	0	SRR8364302
SRR8364303	O1 Inaba	Clinical	2017.391781	0	SRR8364303
SRR8364304	O1 Inaba	Clinical	2017.372603	0	SRR8364304
SRR8364305	O1 Inaba	Clinical	2017.375342	0	SRR8364305
SRR8364306	O1 Inaba	Clinical	2017.372603	0	SRR8364306
SRR8364307	O1 Inaba	Clinical	2017.375342	0	SRR8364307
SRR8364308	O1 Ogawa	Clinical	2016.472678	0	SRR8364308
SRR8364309	O1 Ogawa	Clinical	2013.824658	0	SRR8364309
SRR8364310	O1 Ogawa	Clinical	2013.747945	0	SRR8364310
SRR8364311	O1 Ogawa	Clinical	2013.871233	0	SRR8364311
SRR8364312	O1 Inaba	Clinical	2013.846575	0	SRR8364312
SRR8364313	O1 Ogawa	Clinical	2013.909589	0	SRR8364313
SRR8364314	O1 Ogawa	Clinical	2013.887671	0	SRR8364314
SRR8364315	O1 Ogawa	Clinical	2013.928767	0	SRR8364315
SRR8364316	O1 Ogawa	Clinical	2013.923288	0	SRR8364316
SRR8364317	O1 Ogawa	Clinical	2013.953425	0	SRR8364317
SRR8364318	O1 Ogawa	Clinical	2013.939726	0	SRR8364318
SRR8364319	O1 Inaba	Clinical	2016.191257	0	SRR8364319
SRR8364320	O1 Ogawa	Clinical	2016.448087	0	SRR8364320
SRR8364321	O1 Inaba	Clinical	2016.122951	0	SRR8364321
SRR8364322	O1 Inaba	Clinical	2016.122951	0	SRR8364322
SRR8364323	O1 Inaba	Clinical	2016.122951	0	SRR8364323
SRR8364324	O1 Inaba	Clinical	2016.122951	0	SRR8364324
SRR8364325	O1 Ogawa	Clinical	2017.041096	0	SRR8364325
SRR8364326	O1 Ogawa	Clinical	2017.032877	0	SRR8364326
SRR8364327	O1 Inaba	Clinical	2016.18306	0	SRR8364327
SRR8364328	O1 Ogawa	Clinical	2016.972678	0	SRR8364328
SRR8364329	O1 Ogawa	Clinical	2016.92623	0	SRR8364329
SRR8364330	O1 Inaba	Clinical	2017.008219	0	SRR8364330
SRR8364331	O1 Inaba	Clinical	2016.989071	0	SRR8364331
SRR8364332	O1 Ogawa	Clinical	2017.021918	0	SRR8364332
SRR8364333	O1 Ogawa	Clinical	2017.010959	0	SRR8364333
SRR8364334	O1 Inaba	Clinical	2017.030137	0	SRR8364334
SRR8364335	O1 Inaba	Clinical	2017.024658	0	SRR8364335
SRR8364336	O1 Ogawa	Clinical	2016.497268	0	SRR8364336
SRR8364337	O1 Ogawa	Clinical	2013.610959	0	SRR8364337

Name	Serogroup	Source	Date decimals	Date uncertainty	SRA/ENA accession no.
SRR8364338	O1 Ogawa	Clinical	2013.673973	0	SRR8364338
SRR8364339	O1 Ogawa	Clinical	2013.580822	0	SRR8364339
SRR8364340	O1 Ogawa	Clinical	2013.49863	0	SRR8364340
SRR8364341	O1 Ogawa	Clinical	2013.580822	0	SRR8364341
SRR8364342	O1 Ogawa	Clinical	2013.523288	0	SRR8364342
SRR8364343	O1 Ogawa	Clinical	2013.638356	0	SRR8364343
SRR8364344	O1 Ogawa	Clinical	2013.6	0	SRR8364344
SRR8364345	O1 Ogawa	Clinical	2013.536986	0	SRR8364345
SRR8364346	O1 Inaba	Clinical	2013.536986	0	SRR8364346
SRR8364347	O1 Ogawa	Clinical	2014.863014	0	SRR8364347
SRR8364348	O1 Ogawa	Clinical	2014.810959	0	SRR8364348
SRR8364349	O1 Ogawa	Clinical	2014.906849	0	SRR8364349
SRR8364350	O1 Ogawa	Clinical	2014.90411	0	SRR8364350
SRR8364351	O1 Ogawa	Clinical	2014.958904	0	SRR8364351
SRR8364352	O1 Ogawa	Clinical	2014.906849	0	SRR8364352
SRR8364353	O1 Ogawa	Clinical	2015.079452	0	SRR8364353
SRR8364354	O1 Ogawa	Clinical	2014.975342	0	SRR8364354
SRR8364355	O1 Ogawa	Clinical	2015.189041	0	SRR8364355
SRR8364356	O1 Ogawa	Clinical	2015.134247	0	SRR8364356
SRR8364357	O1 Ogawa	Clinical	2013.953425	0	SRR8364357
SRR8364358	O1 Ogawa	Clinical	2013.953425	0	SRR8364358
SRR8364359	O1 Inaba	Clinical	2017.263014	0	SRR8364359
SRR8364360	O1 Inaba	Clinical	2017.273973	0	SRR8364360
SRR8364361	O1 Inaba	Clinical	2017.172603	0	SRR8364361
SRR8364362	O1 Inaba	Clinical	2017.254795	0	SRR8364362
SRR8364363	O1 Inaba	Clinical	2017.235616	0	SRR8364363
SRR8364364	O1 Inaba	Clinical	2017.241096	0	SRR8364364
SRR8364365	O1 Inaba	Clinical	2017.235616	0	SRR8364365
SRR8364366	O1 Inaba	Clinical	2017.235616	0	SRR8364366
SRR8364367	O1 Inaba	Clinical	2017.279452	0	SRR8364367
SRR8364368	O1 Inaba	Clinical	2017.290411	0	SRR8364368
SRR8364369	O1 Ogawa	Environ	2013.572603	0	SRR8364369
SRR8364370	O1 Ogawa	Clinical	2013.956164	0	SRR8364370
SRR8364371	O1 Ogawa	Environ	2013.572603	0	SRR8364371
SRR8364372	O1 Ogawa	Environ	2013.572603	0	SRR8364372
SRR8364373	O1 Ogawa	Clinical	2014.706849	0	SRR8364373
SRR8364374	O1 Ogawa	Clinical	2014.734247	0	SRR8364374
SRR8364375	O1 Ogawa	Clinical	2014.660274	0	SRR8364375
SRR8364376	O1 Ogawa	Clinical	2014.706849	0	SRR8364376
SRR8364377	O1 Ogawa	Clinical	2014.734247	0	SRR8364377
SRR8364378	O1 Ogawa	Clinical	2014.789041	0	SRR8364378
SRR8364379	O1 Inaba	Clinical	2016.18306	0	SRR8364379
SRR8364380	O1 Inaba	Clinical	2017.317808	0	SRR8364380
SRR8364381	O1 Inaba	Clinical	2017.309589	0	SRR8364381
SRR8364382	O1 Inaba	Clinical	2017.339726	0	SRR8364382
SRR8364383	O1 Inaba	Clinical	2017.320548	0	SRR8364383
SRR8364384	O1 Inaba	Clinical	2017.293151	0	SRR8364384
SRR8364385	O1 Inaba	Clinical	2017.290411	0	SRR8364385
SRR8364386	O1 Inaba	Clinical	2017.30137	0	SRR8364386
SRR8364387	O1 Inaba	Clinical	2017.29863	0	SRR8364387
SRR8364388	O1 Inaba	Clinical	2017.926027	0	SRR8364388
SRR8364389	O1 Inaba	Clinical	2017.843836	0	SRR8364389
SRR8364390	O1 Inaba	Clinical	2015.860274	0	SRR8364390
SRR8364391	O1 Inaba	Clinical	2015.860274	0	SRR8364391
SRR8364392	O1 Inaba	Clinical	2015.860274	0	SRR8364392
SRR8364393	O1 Inaba	Clinical	2015.860274	0	SRR8364393
SRR8364394	O1 Inaba	Clinical	2015.843836	0	SRR8364394
SRR8364395	O1 Inaba	Clinical	2015.843836	0	SRR8364395
SRR8364396	O1 Inaba	Clinical	2015.843836	0	SRR8364396
SRR8364397	O1 Inaba	Clinical	2015.843836	0	SRR8364397
SRR8364398	O1 Inaba	Clinical	2015.863014	0	SRR8364398
SRR8364399	O1 Ogawa	Clinical	2015.860274	0	SRR8364399
SRR8364400	O1 Ogawa	Clinical	2016.606557	0	SRR8364400
SRR8364401	O1 Ogawa	Clinical	2016.510929	0	SRR8364401
SRR8364402	O1 Inaba	Clinical	2017.046575	0	SRR8364402
SRR8364403	O1 Ogawa	Clinical	2017.063014	0	SRR8364403
SRR8364404	O1 Inaba	Clinical	2017.071233	0	SRR8364404
SRR8364405	O1 Inaba	Clinical	2017.084932	0	SRR8364405
SRR8364406	O1 Ogawa	Clinical	2017.117808	0	SRR8364406

Name	Serogroup	Source	Date decimals	Date uncertainty	SRA/ENA accession no.
SRR8364407	O1 Inaba	Clinical	2017.136986	0	SRR8364407
SRR8364408	O1 Inaba	Clinical	2017.136986	0	SRR8364408
SRR8364409	O1 Inaba	Clinical	2017.09589	0	SRR8364409
SRR8364410	O1 Inaba	Clinical	2017.139726	0	SRR8364410
SRR8364411	O1 Inaba	Clinical	2017.139726	0	SRR8364411
SRR8364412	O1 Ogawa	Clinical	2016.775956	0	SRR8364412
SRR8364413	O1 Ogawa	Clinical	2015.282192	0	SRR8364413
SRR8364414	O1 Ogawa	Clinical	2015.358904	0	SRR8364414
SRR8364415	O1 Ogawa	Clinical	2015.517808	0	SRR8364415
SRR8364416	O1 Ogawa	Clinical	2015.731507	0	SRR8364416
SRR8364417	O1 Ogawa	Clinical	2015.780822	0	SRR8364417
SRR8364418	O1 Inaba	Clinical	2015.786301	0	SRR8364418
SRR8364419	O1 Ogawa	Clinical	2015.80274	0	SRR8364419
SRR8364420	O1 Inaba	Clinical	2015.824658	0	SRR8364420
SRR8364421	O1 Inaba	Clinical	2015.824658	0	SRR8364421
SRR8364422	O1 Inaba	Clinical	2015.827397	0	SRR8364422
SRR8364423	O1 Inaba	Clinical	2016.737705	0	SRR8364423
SRR8364424	O1 Inaba	Clinical	2016.122951	0	SRR8364424
SRR8364425	O1 Ogawa	Clinical	2015.920548	0	SRR8364425
SRR8364426	O1 Ogawa	Clinical	2015.920548	0	SRR8364426
SRR8364427	O1 Inaba	Clinical	2015.920548	0	SRR8364427
SRR8364428	O1 Inaba	Clinical	2015.920548	0	SRR8364428
SRR8364429	O1 Inaba	Clinical	2015.920548	0	SRR8364429
SRR8364430	O1 Ogawa	Environ	2013.821918	0	SRR8364430
SRR8364431	O1 Inaba	Clinical	2015.89863	0	SRR8364431
SRR8364432	O1 Inaba	Clinical	2015.920548	0	SRR8364432
SRR8364433	O1 Inaba	Clinical	2015.90137	0	SRR8364433
SRR8364434	O1 Ogawa	Environ	2013.726027	0	SRR8364434
SRR8364435	O1 Inaba	Clinical	2015.90137	0	SRR8364435
SRR8364436	O1 Ogawa	Environ	2013.89863	0	SRR8364436
SRR8364437	O1 Ogawa	Environ	2013.821918	0	SRR8364437
SRR8364438	O1 Inaba	Clinical	2015.884932	0	SRR8364438
SRR8364439	O1 Inaba	Clinical	2015.89863	0	SRR8364439
SRR8364440	O1 Inaba	Clinical	2015.884932	0	SRR8364440
SRR8364441	O1 Inaba	Clinical	2015.884932	0	SRR8364441
SRR8364442	O1 Inaba	Clinical	2015.884932	0	SRR8364442
SRR8364443	O1 Inaba	Clinical	2015.884932	0	SRR8364443
SRR8364444	O1 Inaba	Clinical	2015.865753	0	SRR8364444
SRR8364445	O1 Inaba	Clinical	2015.882192	0	SRR8364445
SRR8364446	O1 Inaba	Clinical	2015.863014	0	SRR8364446
SRR8364447	O1 Inaba	Clinical	2015.863014	0	SRR8364447
SRR8364448	O1 Inaba	Clinical	2017.221918	0	SRR8364448
SRR8364449	O1 Inaba	Clinical	2017.205479	0	SRR8364449
SRR8364450	O1 Inaba	Clinical	2017.19726	0	SRR8364450
SRR8364451	O1 Inaba	Clinical	2017.175342	0	SRR8364451
SRR8364452	O1 Inaba	Clinical	2017.167123	0	SRR8364452
SRR8364453	O1 Inaba	Clinical	2017.164384	0	SRR8364453
VCN3833	O1 Ogawa	Clinical	2022.753425	0	SRR22265444
VCN3834	O1 Ogawa	Clinical	2022.756164	0	SRR22265443
VCO1902542	O1 Ogawa	Clinical	2017.668	0	SAMN33974382
VCO1902543	O1 Inaba	Clinical	2018.369863	0	SAMN33974383
VCO1902544	O1 Inaba	Clinical	2018.372603	0	SAMN33974384
VCO1902545	O1 Inaba	Clinical	2018.375342	0	SAMN33974385
VCO1902546	O1 Inaba	Clinical	2018.372603	0	SAMN33974386
VCO1902547	O1 Inaba	Clinical	2018.375342	0	SAMN33974387
VCO1902548	O1 Inaba	Clinical	2018.378082	0	SAMN33974388
VCO1902549	O1 Inaba	Clinical	2018.380822	0	SAMN33974389
VCO1902552	O1 Inaba	Clinical	2018.389041	0	SAMN33974390
VCO1902553	O1 Inaba	Clinical	2018.391781	0	SAMN33974391
VCO1902554	O1 Inaba	Clinical	2018.394521	0	SAMN33974392
VCO1902555	O1 Inaba	Clinical	2018.405479	0	SAMN33974393
VCO1902556	O1 Inaba	Clinical	2018.408219	0	SAMN33974394
VCO1902558	O1 Inaba	Clinical	2018.427397	0	SAMN33974395
VCO1902560	O1 Inaba	Clinical	2018.443836	0	SAMN33974396
VCO1902561	O1 Inaba	Clinical	2018.175342	0	SAMN33974397
VCO1902562	O1 Inaba	Clinical	2018.30411	0	SAMN33974398
VCO1902563	O1 Inaba	Clinical	2018.317808	0	SRR22265475
VCO1902564	O1 Inaba	Clinical	2018.336986	0	SRR22265474
VCO1902565	O1 Inaba	Clinical	2018.336986	0	SRR22265463

Name	Serogroup	Source	Date decimals	Date uncertainty	SRA/ENA accession no.
VCO1902566	O1 Inaba	Clinical	2018.334247	0	SRR22265452
VCO1902567	O1 Inaba	Clinical	2018.347945	0	SRR22265442
VCO1902568	O1 Inaba	Clinical	2018.350685	0	SRR22265441
VCO1902569	O1 Inaba	Clinical	2018.342466	0	SRR22265440
VCO1902570	O1 Inaba	Clinical	2018.386301	0	SRR22265439
VCO1902571	O1 Inaba	Clinical	2018.413699	0	SRR22265438
VCO1902572	O1 Inaba	Clinical	2018.463014	0	SRR22265437
VCO1902573	O1 Inaba	Clinical	2018.465753	0	SRR22265473
VCO1902574	O1 Inaba	Clinical	2018.468493	0	SRR22265472
VCO1902575	O1 Inaba	Clinical	2018.465753	0	SRR22265471
VCO1902576	O1 Inaba	Clinical	2018.465753	0	SRR22265470
VCO1902577	O1 Inaba	Clinical	2018.468493	0	SRR22265469
VCO1902578	O1 Inaba	Clinical	2018.493151	0	SRR22265468
VCO1902579	O1 Inaba	Clinical	2018.427397	0	SRR22265467
VCO1902580	O1 Inaba	Clinical	2018.430137	0	SRR22265466
VCO1902581	O1 Inaba	Clinical	2018.432877	0	SRR22265465
VCO1902582	O1 Inaba	Clinical	2018.493151	0	SRR22265464
VCO1902583	O1 Inaba	Clinical	2018.438356	0	SRR22265462
VCO1902584	O1 Inaba	Clinical	2018.441096	0	SRR22265461
VCO1902585	O1 Inaba	Clinical	2018.413699	0	SRR22265460
VCO1902586	O1 Inaba	Clinical	2018.50137	0	SRR22265459
VCO1902587	O1 Inaba	Clinical	2018.50411	0	SRR22265458
VCO1902588	O1 Inaba	Clinical	2018.506849	0	SRR22265457
VCO1902589	O1 Inaba	Clinical	2018.356164	0	SRR22265456
VCO1902590	O1 Inaba	Clinical	2018.665753	0	SRR22265455
VCO9902595	O1 Inaba	Clinical	2018.391781	0	SRR22265451
2022V-1171		Clinical	2022.788	0.04246575	SRR23509871
2022V-1172		Clinical	2022.788	0.04246575	SRR23509900
2022V-1174		Clinical	2022.788	0.04246575	SRR23510634
2022V-1175		Clinical	2022.788	0.04246575	SRR23510642
2022V-1176		Clinical	2022.788	0.04246575	SRR23510637
2022V-1177		Clinical	2022.788	0.04246575	SRR23509899
2022V-1178		Clinical	2022.788	0.04246575	SRR23510641
2022V-1179		Clinical	2022.788	0.04246575	SRR23509867
2022V-1180		Clinical	2022.788	0.04246575	SRR23510631
2022V-1181		Clinical	2022.788	0.04246575	SRR23510640
2022V-1182		Clinical	2022.788	0.04246575	SRR23509889
2022V-1183		Clinical	2022.788	0.04246575	SRR23509864
2022V-1184		Clinical	2022.788	0.04246575	SRR23509866
2022V-1185		Clinical	2022.788	0.04246575	SRR23509894
2022V-1186		Clinical	2022.788	0.04246575	SRR23510649
2022V-1187		Clinical	2022.788	0.04246575	SRR23510654

*ENA, European Nucleotide Archive (https://www.ebi.ac.uk/ena); SRA, Sequence Reads Archive (https://www.ncbi.nlm.nih.gov/sra).

Appendix Table 5. Bayes factors for relevant migrations among groups of samples of *Vibrio cholerae* O1, Haiti

Group no.		
From	To	Bayes factor
2	4	8.28272825
2	1	8.02530144
2	3	6.35742392
2	5	5.83860853
1	4	5.6778874
4	3	5.6670122
1	2	5.56500236
1	3	5.39581192
1	5	5.05727025
4	1	4.649288
4	2	4.49052847
4	5	4.35576248
5	1	3.66230118
3	1	3.3638466
5	2	3.30457235
5	4	3.23770477
3	4	3.22048445
3	5	3.21190846
5	3	3.12182361
3	2	3.11627648

Appendix Table 6. Model selection for molecular clock and Bayesian demographic models to infer time-structured phylogeny for *Vibrio cholerae* O1 isolates collected between October 2010 and November 2022, Haiti*

Enforcement of 2022 strains with EnvJ515	$\ln(ML)_{SS}$	$Ln(BF)_{SS}$	$\ln(ML)_{PS}$	$Ln(BF)_{PS}$
No monophyly enforcement				
SC CONST	–1,907.6	3.3	–1,911.1	3.8
RC CONST	–1,904.3		–1,907.3	
SC BSP	–1,903.1	10.3	–1,900.0	4.0
RC BSP	–1,892.8		–1,896.0	
RC CONST	–1,904.3	11.5	–1,907.3	11.3
RC BSP	–1,892.8		–1,896.0	
Monophyly enforcement				
SC CONST (ME)	–1,906.0	6.4	–1,907.8	5.7
RC CONST (ME)	–1,899.6		–1,902.1	
SC BSP (ME)	–1,890.6	–0.2	–1,893.9	–0.6
RC BSP (ME)	–1,890.8		–1,894.4	
RC CONST (ME)	–1,899.6	9.0	–1,902.1	8.3
SC BSP (ME)	–1,890.6		–1,893.9	
Monophyly testing				
SC CONST	–1,907.6	1.6	–1,911.1	3.3
SC CONST (ME)	–1,906.0		–1,907.8	
SC BSP	–1,903.1	12.5	–1,900.0	6.1
SC BSP (ME)	–1,890.6		–1,893.9	
RC BSP	–1,892.8	2.0	–1,896.0	1.6
RC BSP (ME)	–1,890.8		–1,894.4	
RC CONST	–1,904.3039	4.7	–1,907.3	5.2
RC CONST (ME)	–1,899.6		–1,902.1	
RC BSP	–1,892.8	2.2	–1,896.0	2.1
SC BSP (ME)	–1,890.6		–1,893.9	

*Log of marginal likelihood estimate values obtained by stepping-stone and path sampling and are reported for models that used the following as priors: strict or uncorrelated relaxed lognormal (UCLN) molecular clocks and constant, nonparametric Bayesian skyline demographic models with and without monophyly enforcement of strains from 2022 with environmental Ogawa strain EnvJ515. BF, Bayes factor; BSP, Bayesian skyline; CONST, constant; Ln, log; ME, monophyly enforcement; ML, maximum-likelihood; PS, path sampling; SC, strict; SS, stepping-stone.

Appendix Table 7. List of hqSNPs that differentiate the 2022 outbreak strains from EnvJ515 environmental Ogawa strain (using the genome of *Vibrio cholerae* O1 2010EL-1786 as reference strain).

Gene or locus tag	Chr	Genome position	Mutation, nt	Mutation, aa	Protein ID, function	Sample
istA	1	150,844	A→G	Val231Ala	IS 21 family transposes	EnvJ515
moaA	1	571,070	C→A	Thr310Asn	Molybdenum cofactor biosynthesis protein A (hypoxic growth)	2022 outbreak
fadJ	1	594,746	C→T	Ala517Val	Long chain fatty acid metabolism	2022 outbreak
mfd	1	1,503,970	T→C	Val709Ala	SOS response protein in response to sub-inhibitory level of antibiotic concentration	2022 outbreak
HJ37_RS07360†	1	1,658,252	C→CGGCG	Frame shift; 91STOP		2022 outbreak
rpsG	1	2,803,192	A→G	Glu63Glu	30 S ribosomal protein S7	2022 outbreak
Intergenic	2	603,535	G→A	NA		EnvJ515
HJ37_RS17585†	2	731,831	A→G	Gly589Asp		EnvJ515

*A genome of Vibrio cholerae O1 2010EL-1786 was used as the reference strain. aa, amino acid; Chr, chromosome; hqSNPs, high-quality SNPs; ID, identification; NA, not applicable; nt, nucleotide.
†Hypothetical protein.

Appendix Table 8. List of hqSNPs shared among the 2022 outbreak strains and 3 environmental strains used in a study of ancestral origin and dissemination dynamic of reemerging toxigenic *Vibrio cholerae*, Haiti*

Gene or locus tag	Chr	Genome position	Mutation, nt	Mutation, aa	Protein ID, function
acnB	1	15,045	C to T	Asp497Asp	TCA cycle metabolism, regulate antibiotic stress
rimP	1	63,460	C to T	Ile148Ile	Ribosomal maturation factor, acidic stress
Intergenic	1	688,901	C to G	NA	
Intergenic	1	895,812	G to GT	NA	
HJ37_RS04810	1	1,082,883	G to A	Ala101Ala	
HJ37_RS05410	1	1,211,305	G to A	Thr489Ile	
HJ37_RS07435	1	1,675,595	A to G	Lys101Arg	
mnmC	1	1,740,013	T to G	Phe373Val	tRNA 5 methyl amino methyl-2-thiouridine biosynthesis bifurcation protein
Intergenic	1	2,051,378	G to GA	NA	
HJ37_RS09310	1	2,094,531	T to G	Lys279Gln	
HJ37_RS09410	1	2,119,145	G to A	His51His	
Intergenic	1	2,124,160	T to C	NA	
gspG	1	2,372,132	A to C	Phe145Val	Pseudopilin, Type II secretion system
fhuA	1	2,633,809	C to T	Tyr684Tyr	Iron utilization protein
Intergenic	1	2,905,479	AT to A	NA	
HJ37_RS20555†	2	57,250	C to CT	Val16fs	
HJ37_RS20010†	2	111,521	CT to C	Leu45fs	
HJ37_RS19585	2	115,005	A to C	Lys28Asn	
Intergenic	2	208,558	T to TA	NA	
napA	2	315,133	C to T	Ala680Val	Periplasmic nitrate reductase, hypoxic growth
Intergenic	2	501,356	G to T	NA	
Intergenic	2	673,799	C to CG	NA	
HJ37_RS17750	2	767,582	C to A	Glu548Asp	
HJ37_RS18095	2	856,231	G to T	Pro232His	

*Strains used included EnvJ515 environmental Ogawa strain isolated in 2018, Env5156 environmental Ogawa strain isolated in 2016, and Env4303 environmental Ogawa strain isolated in 2015. A genome of *Vibrio cholerae* O1 2010EL-1786 was used as the reference strain. aa, amino acid; Chr, chromosome; hqSNPs, high-quality SNPs; ID, identification; NA, not applicable; nt, nucleotide.
†Pseudogene.

Appendix Table 9. List of hqSNPs shared among 3 environmental strains used in a study of ancestral origin and dissemination dynamic of reemerging toxigenic *Vibrio cholerae*, Haiti*

Gene or locus tag	Chr	Genome position	Mutation, nt	Mutation, aa	Protein ID, function
acnB	1	15,045	C to T	Asp497Asp	TCA cycle metabolism, regulate antibiotic stress
rimP	1	63,460	C to T	Ile148Ile	Ribosomal maturation factor, acidic stress
Intergenic	1	688,901	C to G	NA	
Intergenic	1	785711	C to CA		
Intergenic	1	895,812	G to GT	NA	
HJ37_RS04810	1	1,082,883	G to A	Ala101Ala	
HJ37_RS05410	1	1,211,305	G to A	Thr489Ile	
HJ37_RS06215	1	1410244	GTGG to CGGC	Pro285Ala	
HJ37_RS07435	1	1,675,595	A to G	Lys101Arg	
mnmC	1	1,740,013	T to G	Phe373Val	tRNA 5 methyl amino methyl-2-thiouridine biosynthesis bifurcation protein
Intergenic	1	1802518	CA to C		
Intergenic	1	2029032	T to TA		
Intergenic	1	2,051,378	G to GA	NA	
Intergenic	1	2065191	AG to A		
HJ37_RS09310	1	2,094,531	T to G	Lys279Gln	
HJ37_RS09410	1	2,119,145	G to A	His51His	
Intergenic	1	2,124,160	T to C	NA	
gspG	1	2,372,132	A to C	Phe145Val	Pseudopilin, Type II secretion system
HJ37_RS10885	1	2417048	GCTGTTT to G	Glu67_Thr68del	
fhuA	1	2,633,809	C to T	Tyr684Tyr	Iron utilization protein
Intergenic	1	2753681	CT to C		
Intergenic	1	2,905,479	AT to A	NA	
HJ37_RS14100	2	30347	TACCAGAACCA GAACCAGAAC CAGAACCAGA ACCAGAACCA GAACCAGAAC CAGAACCAGA ACCAGAACCA GAACCAGAAC CAGA to T		Pro345_Glu372del
HJ37_RS20545	2	43877	G to GT	Leu24fs	
Intergenic	2	49152	C to CA		
Intergenic	2	52354	C to CA		
napA	2	315,133	C to T	Ala680Val	Periplasmic nitrate reductase, hypoxic growth
HJ37_RS19900	2	54854	G to A	Ala3Ala	
HJ37_RS20555†	2	57,250	C to CT	Val16fs	
HJ37_RS20010†	2	111,521	CT to C	Leu45fs	
HJ37_RS19585	2	115,005	A to C	Lys28Asn	
Intergenic	2	208,558	T to TA	NA	
Intergenic	2	208558	T to TA		
HJ37_RS15720	v	315133	C to T	Ala680Val	
Intergenic	2	501,356	G to T	NA	
Intergenic	2	556549	T to TG		
Intergenic	2	673789	G to GC		
Intergenic	2	673,799	C to CG	NA	
Intergenic	2	731919	T to TC		
HJ37_RS17750	2	767,582	C to A	Glu548Asp	
HJ37_RS18095	2	856,231	G to T	Pro232His	

*Strains used included EnvJ515 environmental Ogawa strain isolated in 2018, Env5156 environmental Ogawa strain isolated in 2016, and Env4303 environmental Ogawa strain isolated in 2015. A genome of *Vibrio cholerae* O1 2010EL-1786 was used as the reference strain. aa, amino acid; Chr, chromosome; hqSNPs, high-quality SNPs; ID, identification; NA, not applicable; nt, nucleotide.
†Pseudogene.

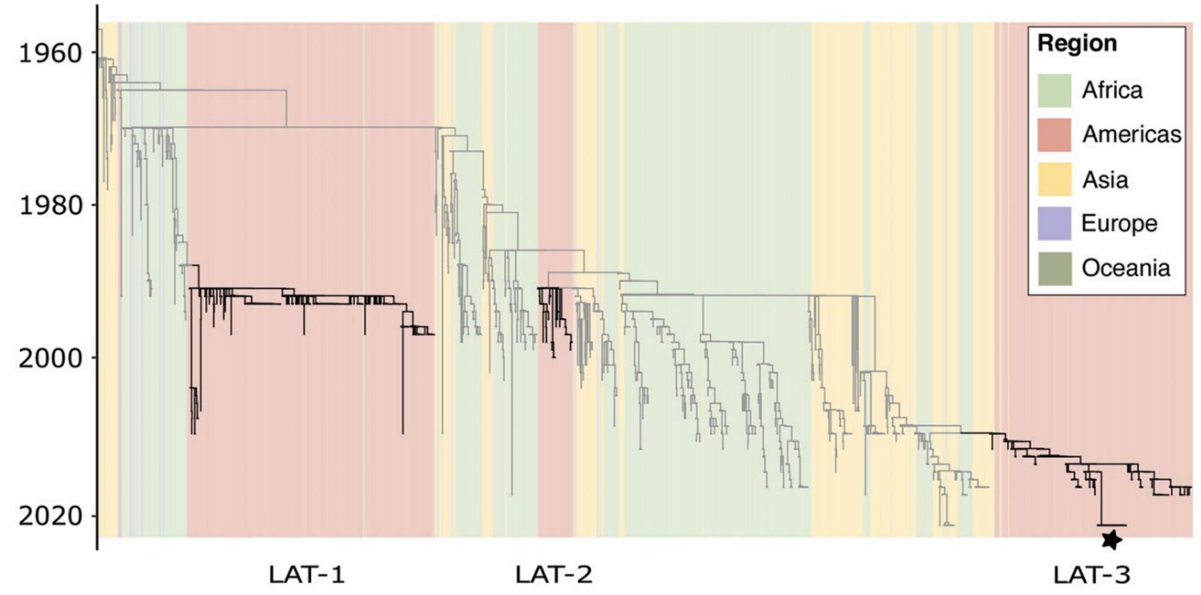

Appendix Figure 1. Maximum-likelihood phylogeny of global *Vibrio cholerae* O1 strains isolated during 1937–2022. The tree was inferred from genome-wide high-quality SNPs, with branches scaled in time using TreeTime (https://github.com/neherlab/treetime). Heatmap denotes major geographic region of collection, according to the colors in the legend. The Latin American clades are colored in cyan, green, and purple, respectively. LAT-1 includes strains introduced into Peru in 1991 that spread across South America; LAT-2, strains introduced into Mexico in 1991, and LAT-3, the strains of the 2010–2022 Haiti epidemic, with the monophyletic group of the 2022 sequences highlighted by a star.

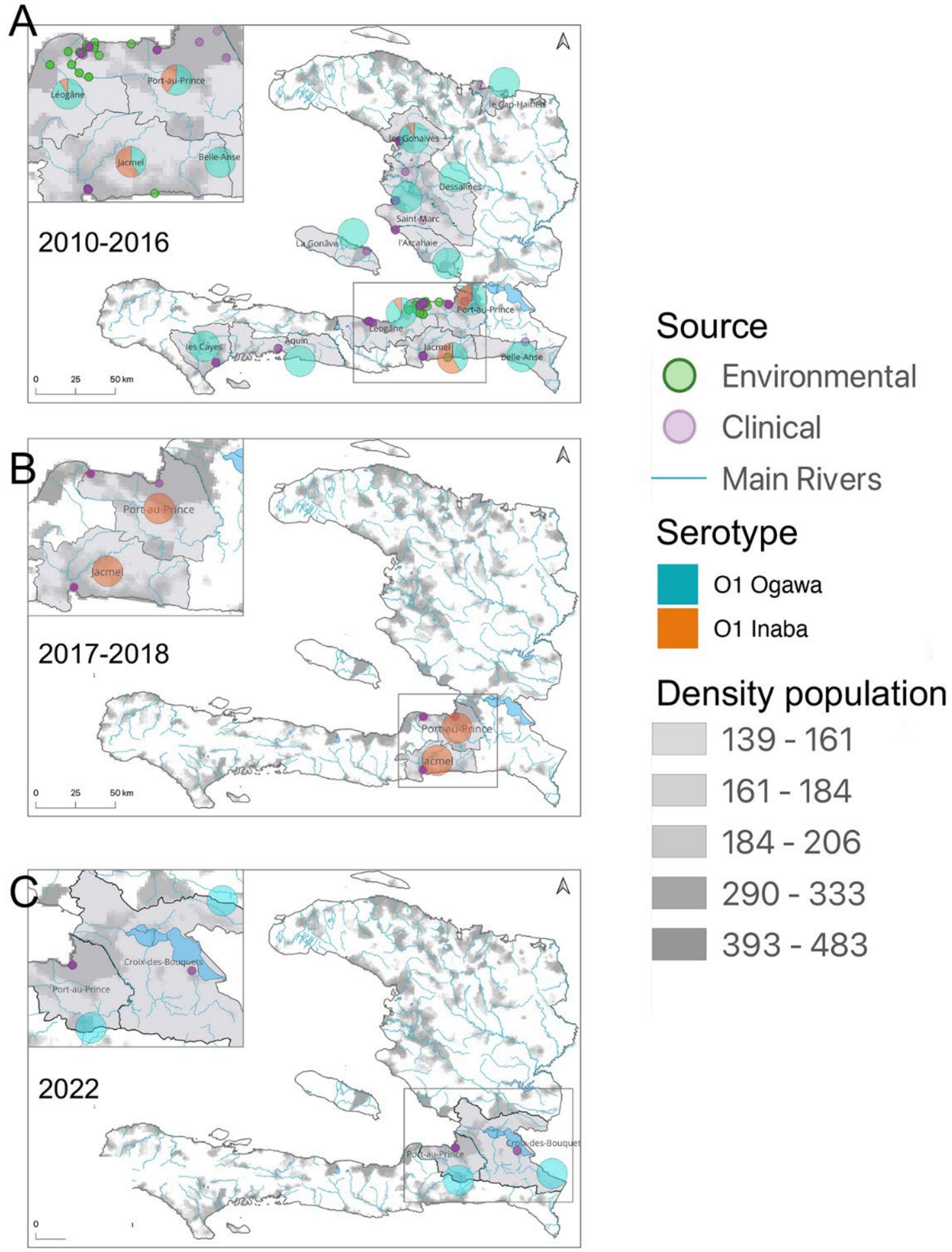

Appendix Figure 2. Toxigenic *Vibrio cholerae* O1 strains sampled in Haiti from 2010–2022. Haiti maps with population density show departments where sampling occurred. A) 2010– 2016; B) 2017–2018; C) 2022. Serotype – Ogawa (blue) and Inaba (orange) – and the source – environmental (green) or clinical (purple) – of all the strains collected, sequenced, and used in this study are denoted.

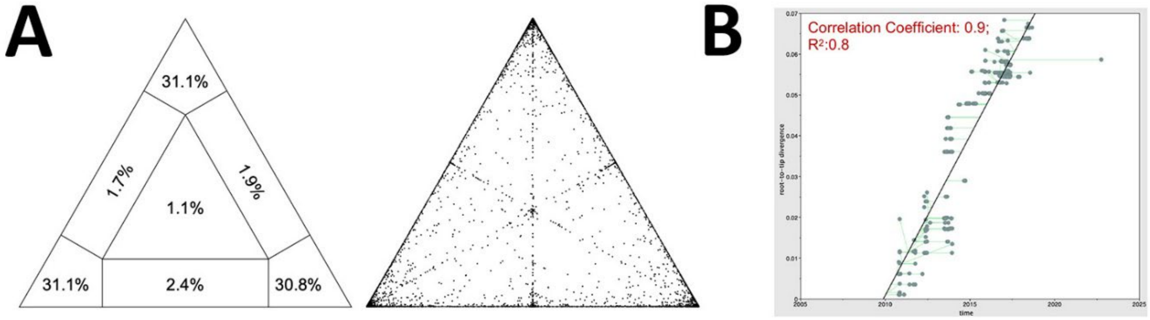

Appendix Figure 3. Phylogenetic quality of the datasets used in a study of ancestral origin and dissemination dynamic of reemerging toxigenic *Vibrio cholerae*, Haiti. A) Presence of phylogenetic signal was evaluated by likelihood mapping checking for alternative topologies (tips), unresolved quartets (center), and partly resolved quartets (edges) for the dataset. B) Linear regression of root-to-tip genetic distance within the maximum-likelihood phylogeny against sampling time for each taxon: temporal resolution was assessed using the slope of the regression, with positive slope indicating sufficient temporal signal.
